# An SPM-Enriched Marine Oil Supplement Shifted Microglia Polarization toward M2, Ameliorating Retinal Degeneration in *rd10* Mice

**DOI:** 10.3390/antiox12010098

**Published:** 2022-12-30

**Authors:** Lorena Olivares-González, Sheyla Velasco, Idoia Gallego, Marina Esteban-Medina, Gustavo Puras, Carlos Loucera, Alicia Martínez-Romero, María Peña-Chilet, José Luis Pedraz, Regina Rodrigo

**Affiliations:** 1 Group of Pathophysiology and Therapies for Vision Disorders, Príncipe Felipe Research Center (CIPF), 46012 Valencia, Spain; 2 NanoBioCel Group, Laboratory of Pharmaceutics, School of Pharmacy, University of the Basque Country (UPV/EHU), 01006 Vitoria-Gasteiz, Spain; 3 Biomedical Research Networking Center in Bioengineering, Biomaterials and Nanomedicine (CIBER-BBN), Health Institute Carlos III, 01006 Vitoria-Gasteiz, Spain; 4 Bioaraba, NanoBioCel Research Group, 01006 Vitoria-Gasteiz, Spain; 5 Computational Medicine Platform, Andalusian Public Foundation Progress and Health-FPS Hospital Virgen del Rocío, 41013 Seville, Spain; 6 Systems and Computational Medicine Group, Institute of Biomedicine of Seville, IBiS, University Hospital Virgen del Rocío/CSIC/University of Seville, 41013 Seville, Spain; 7 Cytomic Unit, Príncipe Felipe Research Center (CIPF), 46012 Valencia, Spain; 8 Bioinformatics in Rare Diseases (BiER), Centro de Investigación Biomédica en Red de Enfermedades Raras (CIBERER), FPS, Hospital Virgen del Rocio, 41013 Seville, Spain; 9 Biomedical Research Networking Center in Rare Diseases (CIBERER), Health Institute Carlos III, 28029 Madrid, Spain; 10 Department of Physiology, University of Valencia (UV), 46100 Burjassot, Spain; 11 Department of Anatomy and Physiology, Catholic University of Valencia San Vicente Mártir, 46001 Valencia, Spain; 12 Joint Research Unit on Endocrinology, Nutrition and Clinical Dietetics UV-IIS La Fe, 46026 Valencia, Spain

**Keywords:** inherited retinal dystrophies, retinitis pigmentosa, inflammation, microglia, specialized pro-resolving mediators, oxidative stress

## Abstract

Retinitis pigmentosa (RP) is the most common inherited retinal dystrophy causing progressive vision loss. It is accompanied by chronic and sustained inflammation, including M1 microglia activation. This study evaluated the effect of an essential fatty acid (EFA) supplement containing specialized pro-resolving mediators (SPMs), on retinal degeneration and microglia activation in *rd10* mice, a model of RP, as well as on LPS-stimulated BV2 cells. The EFA supplement was orally administered to mice from postnatal day (P)9 to P18. At P18, the electrical activity of the retina was examined by electroretinography (ERG) and innate behavior in response to light were measured. Retinal degeneration was studied via histology including the TUNEL assay and microglia immunolabeling. Microglia polarization (M1/M2) was assessed by flow cytometry, qPCR, ELISA and histology. Redox status was analyzed by measuring antioxidant enzymes and markers of oxidative damage. Interestingly, the EFA supplement ameliorated retinal dysfunction and degeneration by improving ERG recording and sensitivity to light, and reducing photoreceptor cell loss. The EFA supplement reduced inflammation and microglia activation attenuating M1 markers as well as inducing a shift to the M2 phenotype in *rd10* mouse retinas and LPS-stimulated BV2 cells. It also reduced oxidative stress markers of lipid peroxidation and carbonylation. These findings could open up new therapeutic opportunities based on resolving inflammation with oral supplementation with SPMs such as the EFA supplement.

## 1. Introduction

Retinitis pigmentosa (RP) is the most common inherited retinal dystrophy that causes progressive loss of vision [1]. It is a rare disease (1/4000), mainly associated with photoreceptor (PR, rods and cones) dysfunction and their loss that eventually leads to blindness. RP has a high genetic (more than 125 genes) and clinical heterogeneity, which makes it difficult to find a correct treatment. In humans, no sex predilection exists except for X-linked RP, which is mainly expressed in males. Typical symptoms of RP are the loss of night vision during adolescence, peripheral vision in adulthood, and central vision in the later stages of the disease. Genetic mutations cause a progressive loss of rods in the early stages of RP. After the death of the rods, compounds such as free radicals and inflammatory molecules are released into the environment, generating a hostile microenvironment in the retina that compromises the survival of the cones [2]. The inflammatory microenvironment seems to play a vital role in the pathogenesis and progression of RP.

Parallel to the disease progression, RP patients and *rd10* mice, a model of autosomal recessive RP, exhibit a sustained and chronic inflammatory process [3,4]. Microglia activation is a hallmark of RP. Activated microglia cells mainly polarize into two phenotypes: an M1 pro-inflammatory phenotype (classical activation) and an M2 anti-inflammatory phenotype (alternative activation). M1 microglia are typified by the high production of pro-inflammatory cytokines such as interleukin (IL)1β, tumor necrosis factor alpha (TNFα), and reactive nitrogen and oxygen species (RNS and ROS), activating endothelial cells and inducing the recruitment of other immune cells. M2 microglia, conversely, are commonly associated with tissue remodeling and repair, efficient scavenging of debris (phagocytosis of apoptotic cells, efferocytosis) and resolution of inflammatory responses [5].

The retina is rich in lipids derived from essential omega 3 and 6 (ω-3 and ω-6) long-chain polyunsaturated fatty acids (LC-PUFAs), which are critical for retinal functions. Alpha-linolenic acid (ω-3) is the dietary precursor to eicosapentaenoic acid (EPA) and docosahexaenoic acid (DHA), whereas linoleic acid (ω-6) is the dietary precursor to arachidonic acid (AA). LC-PUFAs are metabolized to pro- and anti-inflammatory metabolites through cyclooxygenases (COXs, COX1 and COX2), cytochrome P450 oxidases (CYPs) and lipoxygenase (ALOXs). These metabolites cause innate and acquired immune responses that would affect retinal inflammation in chronic retinal diseases. In general, COXs convert LC-PUFAs into prostaglandins (PGs, through COX1), thromboxanes and resolvins (TX and RV through COX2) and CYPs in autocoids and epoxyeicosatrienoic acids (e.g., 20-HETE), with anti-inflammatory properties in different vasculature beds [6]. On the other hand, ALOXs catalyzes LC-PUFAs to form the bioactive lipid SPMs [7].

**Figure 1:**
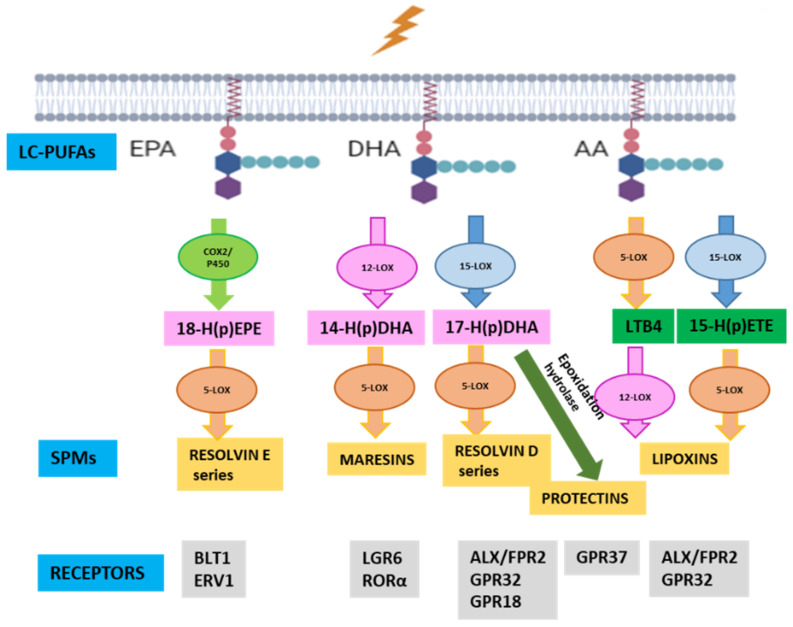
Specialized pro-resolving mediator (SPM) synthesis. SPMs are produced sequentially through action of specific enzymes, cyclooxygenases (COX), cytochrome P450 and lipoxygenases (LOX), from long-chain polyunsaturated fatty acids (LC-PUFA), including eicosapentaenoic acid (EPA), decosahexaenoic acid (DHA) and arachidonic acid (AA). Each SPM executes its functions by binding to a specific receptor.

Specialized pro-resolving mediators (SPMs) derived from EPA, DHA and AA, including lipoxins (LX), RV, protectins (PD), and maresins (MaR), can terminate and resolve inflammation leading to tissue repair and regeneration. SPMs are naturally synthesized in immune cells [8,9]. Photoreceptors (PRs) have the highest DHA content in the body. The 5-, 15- and 15B-lipoxygenases (ALOX5, ALOX15 and ALOX15B) are the rate-limiting enzymes responsible for generating SPMs [10] (Figure 1). It is suggested that SPMs play a critical role in modulating the crosstalk between neurons and glial cells [11]. The main functions of SPMs include inhibiting pro-inflammatory cytokine release, oxidative stress, NFkβ activation, neutrophil infiltration, and cell death of neurons and microglia. In addition, they promote phagocytosis of apoptotic neutrophils (efferocytosis), expression of Nrf2, and M2 macrophage/microglia polarization [12,13] Pro-resolving activities of SPMs occur through the activation of a receptor of the family of G-protein-coupled receptors (GPCRs) including FPR2/ALX, GPR32, GPR18, BLT1, GPR37, LGR6 etc. (Figure 1). Furthermore, retinal elongases, especially ELOVL4, synthetize very-long-chain PUFAs (VLC-PUFAs) [14] from DHA, which are converted in elovanoids (PD analogues) in response to oxidative stress protecting PRs and RPE cells [15].

Currently, there are very little published data related to SPMs and RP progression. Lu et al. showed an upregulation of *Alox5* and *Alox15* in rd1 retinas, a murine model of RP, in the early stages and a downregulation of *Alox15* in the end stages of retinal degeneration. In addition, a significant downregulation of AA and DHA was described in *rd10* mouse retinas, another murine model of RP. The authors suggested that these fatty acids could be essential for PR survival and proposed to design nutritional supplements [16].

In this study, we used the essential fatty acid (EFA) supplement named LIPINOVA^®^. LIPINOVA® is a bioactive formulation consisting of an enriched marine lipid fraction containing EPA, DHA and the precursors 17-HDHA, 14-HDHA and 18-HEPE, that could promote resolution of inflammation during RP progression. Currently, many dietary supplements are composed of variable marine sources of n-3 LC-PUFA oils, but they are not enriched with SPMs, as is LIPINOVA^®^. To date, LIPINOVA^®^ has demonstrated its safety and efficacy in some clinical studies [17,18] and more recently, in preclinical studies [19].

**Figure 2:**
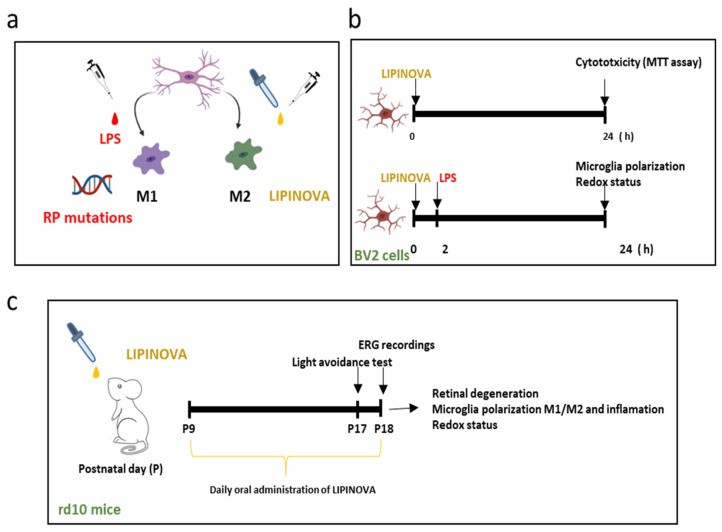
Schematic diagram of the experimental studies to evaluate the actions of the essential fatty acid (EFA) supplement named LIPINOVA^®^ on microglia polarization and retinal degeneration in *rd10* mice. Exogenous LPS administration (1 µg/mL) in BV2 cells or mutations in PDE6 in *rd10* mice induce M1-like microglia phenotype (**a**). We assessed the EFA supplement effect in in vitro (**b**) and in vivo (**c**) experiments. For in vitro studies, BV2 cells were pre-incubated with EFA supplement for 2 h prior adding LPS. After 24 h, BV2 cells were collected for posterior analysis. For in vivo studies, *rd10* mice were treated with EFA supplement orally from postnatal day (P)9 to P18 before analyzing retinal degeneration and inflammation.

In the present study we analyzed the temporal profile of key enzymes involved in SPMs and elovanoid synthesis in *rd10* mouse retinas, and evaluated the effect of an EFA supplement containing SPM precursors on microglia polarization in BV2 cells and retinal degeneration in *rd10* mice (Figure 2).

## 2. Materials and Methods

### 2.1. Mechanistic Modeling of the Relevance of ELOVL4 and ALOX5 on an RP Mechanistic Map

The KEGG pathways are a collection of pathway maps representing our knowledge of the molecular interaction, reaction and relationship networks for metabolism including lipids, genetic information, human diseases, drug development and cellular processes, among others. The impact of clinically relevant target genes over the KEGG pathways associated with RP was evaluated. Then, genotype-tissue expression (GTEx) for normal tissue expression values and the HiPathia R Bioconductor package were used to calculate normal cellular activities as is described by the authors in [20]. The methodology explained by Loucera et al., 2020 [21] was used to infer the possible regulatory capabilities of the targets ALOX5 and ELOVL4 (KDTs, known drug targets) over the disease map we used. Briefly, a multi-output random forest [22] was applied, which built a forest of multi-output decision trees given a training set in the form (X, Y), where X represented the features (normalized gene expression) and Y represented the response (Hipathia signaling activation values, one output per circuit that conformed to the map). Once the model was fitted to a dataset, its behavior was explained by computing the Shapley additive explanations (SHAP) [23]. Each sample was decomposed into the contributions of each feature to every output circuit, then each gene (KDT) was ranked by aggregating the absolute values of the contributions across the samples. After ranking all of the KDTs for each circuit, the 0.95 quantile filtered by the R^2^ (coefficient of determination) score obtained for any given circuit using a convex formula was obtained. When the circuit was perfectly predicted (r = 1), the top 5% of genes as ranked by the mean absolute Shapley value were selected. In contrast, when the predictability was not good enough, the percentage of genes selected was decreased. The stability test proposed by Nogueira et al., 2017 [24] was applied to confirm that the model was accurate and the features selected were stable. The test provided a 95% confidence interval for the stability of the feature selection procedure with an effect size. The R/Bioconductor 3.13 on R v4.1.0. software was used for analyses of annotation, enrichment and visualization.

### 2.2. EFA Supplement Manufacturing Process

As described above, we use LIPINOVA^®^ as the EFA supplement, an SPM-enriched marine oil supplement containing PUFAs, among other fatty acids, from Solutex Corporation, Alcobendas, Madrid, Spain. It has demonstrated pro-resolving activity covered by the patent family PCT/US2013/040313. We determined the composition of fatty acids according to the European Pharmacopoeia (Section 2.4.29, Composition of fatty acids in oils rich in omega-3 acids) [25], expressed as g in 100 g of oil (Table 1). The concentrations of the SPM precursors were determined as previously described by Strassburg, K, 2012 [26], with modifications. Briefly, samples were analyzed by liquid chromatography (Agilent 1260, San Jose, CA, USA) coupled to electrospray ionization on a triple quadrupole mass spectrometer (Agilent 6410, San Jose, CA, USA). For analysis, 10 μL of the extract was injected. The auto sampler was cooled at 10 °C. Chromatographic separation was achieved on a ZORBAX Eclipse Plus (2.1 × 50 mm, 1.8 µm particles; Agilent) column using a flow rate of 0.5 mL/min at room temperature during an 11 min gradient (0–4 min 58% B, 4–10 min B to 100%, 10–10.5 min to 58% B, 10.5–11 min to 58% B), while using the solvents A, 0.01 % acetic acid in water; and B, 0.01% acetic acid in methanol. Electrospray ionization was performed in the negative ion mode using N_2_ at a pressure of 35 psi for the nebulizer with a flow of 10 L/min and a temperature of 300 °C The sheath gas temperature was 350 °C with a flow rate of 8 L/min. The capillary was set at 4000 V. For fatty acid analysis, the extract contained 17-HDHA (182.6 mg/kg), 18-HEPE (200.7 mg/kg) and 14-HDHA (188.2 mg/kg).

For HPLC-QQQ analysis, methanol HPLC grade (99%), n-hexane (96%) and glacial acetic acid (HPLC grade) were purchased from VWR (Radnor, PA, USA). Methyl formate, was purchased from ACROS, Thermo Fischer (Geel, Belgium). Butylated hydroxytoluene (BHT, 99%) was obtained from Sigma Aldrich (San Luis, MO, USA). Standards 17-HDHA (100 mg/L) and 18-HEPE (100 mg/L) were purchased from Cayman Chemicals (Ann Arbor, MI, USA). The analysis is shown in Table 1.

### 2.3. Cell Culture of 661W and BV2

The cell line 661W are PR cells were kindly provided by Dr. Muayyad Al-Ubaidi (University of Oklahoma Health Sciences Center). Cells were grown in Dulbecco’s Modified Eagle Medium/Nutrient Mixture F-12 Ham (#11320033 DMEM:F12; Gibco Thermo Fisher Scientific, Madrid, Spain) supplemented with fetal bovine serum (#26140079, Gibco, Thermo Fisher Scientific, Madrid, Spain), 100 units/mL penicillin/100 µg/mL streptomycin (#15140122, Gibco, Thermo Fisher Scientific, Madrid, Spain) and 0.004% β-mercaptoethanol (#M7154, Sigma-Aldrich, Madrid, Spain). Cells were incubated at 37 °C in 5% CO_2_ humidified atmosphere (#3121, Thermo Electron Corporation, Waltham, MA, USA). For cytotoxicity assays, 661W cell lines were seeded in 96-well plates, at a concentration of 150,000 cells/mL.

The BV2 cells were kindly provided by Dr. Guerri (Principe Felipe Research Center, Valencia). Cells were grown in Dulbecco’s Modified Eagle Medium (#11995-040, DMEM; Gibco, Thermo Fisher Scientific, Madrid, Spain) supplemented with 10% fetal bovine serum (#26140079, Gibco, Thermo Fisher Scientific, Madrid, Spain) and 100 units/mL penicillin/100 µg/mL streptomycin (#15140122, Gibco, Thermo Fisher Scientific, Madrid, Spain). Cells were incubated at 37 °C in 5% CO_2_ humidified atmosphere (#3121, Thermo Electron Corporation, Waltham, MA, USA). For the different experiments, BV2 cells were seeded at 150,000 cells/mL in different plates (6, 8, 24 and 96 wells). BV2 cells were used for cell viability studies, internalization studies, and for analyzing the action of the EFA supplement on microglia polarization (Figure 2a).

BV2 cells were activated in the following manner: 24 h after seeding, BV2 cells were pre-incubated in the EFA supplement for 2 h prior to adding 1 µg/mL of lipopolysaccharide (LPS) to induce microglia activation (M1 phenotype). After 24 h, BV2 cells were collected for posterior analysis.

### 2.4. Cell Viability

The 661W and BV2 cells were grown and then treated with different concentrations of EFA supplement (ranging from 0.0000047 to 0.94 mg/mL) for 24 h. To test cell viability, 100 μL of 3-(4,5-dimethylthiazol-2-yl)-2,5-diphenyltetrazolium bromide (#M5655, MTT; Sigma-Aldrich, Madrid, Spain) from a 5 mg/mL of 0.1M PBS was added to the wells, and the cells were incubated for two hours at 37 °C. Then, the media were removed, and the formazan precipitate was dissolved in 100 µL dimethyl sulfoxide (DMSO) (#D2650, Sigma-Aldrich, Madrid, Spain). The absorbance was read at 550 nm in a Multiskan SkyHigh Microplate Spectrophotometer (Thermo Fisher Scientific, Madrid, Spain). The viability of 661W or BV2 cells was expressed as the percentage of untreated cells (100% of viability) (Figure 2b).

### 2.5. Synthesis of Coumarin-6-Loaded Nanostructured Lipid Carriers (NLC) and the Internalization Study

Coumarin-6-loaded NLCs (6Cou-NP) were elaborated using the hot-melt homogenization technique. Briefly, the lipid core consisted of Precirol ATO 5 (Gattefossé SA, Madrid, Spain) and Miglyol 812N (IOI Oleochemicals, Hamburg, Germany) with coumarin 6 ((3-(2-benzothiazolyl)-*N,N*-diethylumbelliferylamine, 3-(2-benzothiazolyl)-7-(diethylamino)coumarin, Sigma-Aldrich, Madrid, Spain) at 0.05% (*w*/*w*). For the aqueous solution, 1.3% (*w*/*v*) Tween® 80 (Panreac Chemicals, Barcelona, Spain) and 0.6% (*w*/*v*) Poloxamer 188 (BASF, Ludwigshafen, Germany) in MilliQ water were employed. Both phases were heated above the melting temperature of Precirol ATO5 solid lipid, mixed and emulsified by sonication at 50 W for 30 s. Coumarin-6-loaded NLCs were gradually cooled to allow the re-crystallization of lipids and green fluorescent nanoparticle formation. Then, ultrafiltration-washing steps were carried out at 600 g using Amicon^®^ devices with a molecular weight cut-off of 100 kDa (Merck Millipore, Darmstadt, Germany). Coumarin-6-loaded NLCs were freeze–dried (Telstar Lyobeta freeze–dryer, Terrassa, Spain) with 15% trehalose (*w*/*w*) as the cryoprotectant. 

For the internalization study, BV2 cells were seeded at an initial density of 250,000 cells/mL in 24-well plates for flow cytometry and at 25,000 cells/mL in 8-well chamber slides for microscopy. Then, 24 h after seeding, the EFA supplement was added and the cells were incubated for 24 h. For flow cytometry, cells were incubated with 6Cou-NP (12.5 µg/mL) for 15, 30 and 60 min. Cells were centrifuged at 300 g for 3 min and resuspended in the culture medium with the EFA supplement and 1µg/mL propidium iodide (PI). Flow cytometry of the samples was performed using a CytoFLEX S (Beckman Coulter Life Sciences, Indianapolis, IN, USA). For microscopy, cells were fixed in filtered 4% paraformaldehyde (PFA, #158127, Sigma-Aldrich, Madrid, Spain) in PBS for 10 min at room temperature. Then, they were counterstained with DAPI, mounted in Fluoromount-G (#0100-01, South-ern Biotechnology, Birmingham, AL, USA) and observed under a fluorescence microscope (100× magnification, LEICA, DMi8, Leica Microsystems CMS GmbH, Mannheim, Germany) at 22 °C.

### 2.6. Immnunocytochemistry

BV2 cells were fixed in filtered 4% PFA in PBS for 10 min at room temperature. Slides were incubated in a blocking solution containing 5% normal goat serum, 1% bovine serum albumin and 0.25% Triton X-100 (#A1388, Panreac Applichem, Darmstadt, Germany) for one hour. They were later incubated with a primary antibody against arginase-1 (ARG1) (1:200, # 16001-1-AP, ProteinTech, Manchester, UK) and TNFα (1:250, # 60291-1-Ig, ProteinTech, Manchester, United Kingdom) overnight at 4 °C. Then, the slides were incubated with the fluorescence-conjugated secondary antibodies Alexa Fluor 488 or 647 (1:400, #A-11001, #A-21235, Invitrogen, Life Technologies, Madrid, Spain) for one hour at room temperature. After labelling and counterstaining with DAPI, the slides were mounted in Fluoromount-G, and observed under a fluorescence microscope (100X magnification). Slides without the primary antibody served as a negative control.

### 2.7. Animals and Treatment

We used the *rd10* mouse as an animal model of autosomal recessive RP. C57Bl6/6J mice were used as a control since they have the same genetic background as *rd10* mice. The mice were kept under a 12 h light/dark cycle, controlled humidity and temperature in the Animal Facility of the Research Center Principe Felipe (CIPF). The cages were placed on the bottom shelf of an IVC rack to maintain a light illumination of 115 7 lux (95% CI: 98–131). Mothers were fed, as described above, with a standard chow diet and water ad libitum [27]. At least seven animals/group were used for each type of study (electroretinography, behavioral tests, microglia analysis, histological evaluation, redox status, and gene expression).

*Rd10* pups received the EFA supplement at a dose of 2.82 mg/g body weight (bw) by gavage daily (3 µL/g) for nine days starting at postnatal day (P)9. During this period bw ranged from 5 g to 9 g depending on the litter size and the postnatal day. Therefore, mice received approximately 14.1 mg to 25.4 mg of the EFA supplement daily (Figure 2c). As previously described [27], this method allowed the administration of an exact dose. The estimated dose of each fatty acid is shown in Table 2. Both males and females were used. Pups were euthanized by cervical dislocation at P18. No apparent side effects were detected in mice treated with the lipid extract. For redox, cytokine and gene expression studies, retinas were isolated, placed immediately into the appropriate buffer and stored at −80 °C. For flow cytometry, freshly isolated retinas were used. Finally, for histological studies, eyes were fixed and treated as described below.

### 2.8. Electroretinogram 

The global function of the retina was examined by full-field electroretinogram (ERG) under a dim red light in a dark room. The experiments were carried out on 10 control mice (wild-type C57BL/6J), 17 untreated *rd10* mice and 16 *rd10* mice treated with the EFA supplement at postnatal day 18. Mice were kept in darkness for 12 h prior to the experiments for correct adaptation. The mice were anesthetized with inhaled sevoflurane before the procedure, and we used a drop of tropicamide 0.5%/phenylephrine hydrochloride 0.5% to dilate the pupils. To maintain body temperature at 38 °C, the animals were kept on a heated table. 

Responses to light flashes were amplified, averaged and stored using a RetiScan–RetiPort electrophysiology unit (Roland Consult, Germany). Responses were collected simultaneously from both eyes. Before ERGs were recorded, impedance and baseline tests were performed; the latter was performed to evaluate the noise level in the environment. The scotopic responses, which primarily reflect rod function, were evoked by flashes with intensities ranging from 0.0003 to 25 cd/m^2^. For each light intensity, a series of ten ERG responses were averaged and the interval between light flashes was adjusted to 10 s to allow response recovery. The ERG is composed of an initial negative component (a-wave) and subsequent positive peak (b-wave) evoked by light stimulation. The a-wave amplitude was measured from the baseline to the first negative peak, and the b-wave amplitude was measured from the a-wave trough to the subsequent largest positive peak. Retinal activity was represented as the mean ± standard error of the mean of b-wave amplitude for each light flash under scotopic conditions. 

### 2.9. Light/Dark Transition Test

A light/dark transition test was carried out according to previous studies [28]. It was performed in 10 control mice (wild-type C57BL/6J), 15 untreated *rd10* mice, and 16 *rd10* mice treated with the EFA supplement at P17. The apparatus consisted of an acrylic cage (40 × 40 × 44 cm) divided into two sections of equal size by a partition with an opening (ANY-box light/dark #63000, Stoelting Co., Wood Dale, USA). One chamber had transparent walls and was brightly illuminated (800 lx) by a light above the ceiling of the chamber, and the other chamber had black walls and was dark. Mice were placed into the bright chamber and allowed to move freely between the two chambers for 5 min. A camera tracked X- and Y-movements during this period. The time spent in each chamber and the total number of transitions were analyzed using the ANY-maze software (version 7.16, Stoelting Europe, Churchtown, Dublin, Ireland).

### 2.10. Retinal Histology

To obtain retinal sections, the eyes were rapidly removed and fixed in filtered 4% PFA for two hours at room temperature and cryoprotected in a sucrose gradient (15–20–30%). Eyes were frozen and embedded in OCT, and 10 μm sections were cut in a cryostat (Leica CM1900, Nussloch, Germany). We used DAPI (#D9542, Sigma-Aldrich, Madrid, Spain) to determine the number of nuclei in the outer nuclear layer (ONL). To evaluate cell death, the TUNEL assay was used, as previously described [29].

Immunofluorescent staining procedures were performed in 10 μm cryosections. Sections were post-fixed in filtered 4% PFA in 0.1 M phosphate buffer pH 7.4 for 15 min at room temperature. Sections were pre-treated with citrate buffer pH 6.0 for epitope retrieval and incubated in blocking solution containing 5% normal goat serum, 1% bovine serum albumin and 0.25% Triton X-100 (#A1388, Panreac Applichem, Darmstadt, Germany) for one hour. Next, the samples were incubated with the primary antibody against ionized calcium-binding adaptor protein-1 (Iba1) (1:300, #019-19741, Wako Pure Chemical Industries Ltd., Osaka, Japan), CD206 (1:75, #AF2535, R&D Systems, Canada, USA), and glial fibrillary acidic protein (GFAP) (1:400, #G3893, Sigma-Aldrich, Madrid, Spain), overnight at 4 °C. Then, sections were incubated with the fluorescence-conjugated secondary antibodies Alexa Fluor 488 or 647 (1:400, #A-11001, #A-21235, Invitrogen, Life Technologies, Madrid, Spain) for one hour at room temperature. After labelling and counterstaining with DAPI, the sections were mounted in Fluoromount-G, and observed under an SP5 confocal microscope at 22 °C. Eight left and right retinas were analyzed from each group. Slides without the primary antibody served as the negative control.

### 2.11. Microscopy and Quantification

Immunocytochemical staining of BV2 cells was visualized under a fluorescence microscope (100× magnification, LEICA, DMi8, Leica Microsystems CMS GmbH, Mannheim, Germany) belonging to the Microscopy Unit of the CIPF (Valencia, Spain). Retinal sections were examined under a confocal microscope (40× magnification, Leica TCS SP5 Confocal microscope, Leica Microsystems CMS GmbH, Mannheim, Germany) belonging to the Microscopy Unit of the IIS-La Fe (Valencia, Spain) using a spatial resolution of 512 × 512.

The stacks were taken at 40× magnification with an acquisition rate of 16 frames per second. The zoom factor used was 4.0. The Leica LAS AF imaging software was used. Auto-florescence or background noise was detected by negative controls (samples without the primary antibody). Sequential acquisition was used to avoid interference between fluorophores, in the case of double stains. The counting of the number of ONL nuclei, the number of TUNEL+ cells, the positive marking for CD206 and Iba1 and the integrated density of GFAP were made using the ImageJ open-source software (version 1.53, ImageJ, U.S. National Institutes of Health, Bethesda, Maryland, USA). The final images were processed using Adobe Photoshop 10 software (Adobe Systems Inc., San Jose, CA, USA). As the degenerative process in the *rd10* model varies in different retinal locations, we performed several measurements across the entire retina (from the nasal to the temporal retina) for each mouse. At least eight entire retinas were analyzed per experimental group.

The number of Iba1- or/and CD206-positive cells was represented as the ratio between the number of Iba1- or/and CD206-positive cells per retinal area or retinal section. Microglial activation was measured as previously described [30]. Corrected fluorescence of GFAP was quantified as previously described [31]. Iba1-, CD206-positive cells, microglial migration index and the corrected fluorescence of GFAP were quantified from six non-adjacent sections of at least eight retinas for each experimental group.

### 2.12. Gene Expression

Total RNA was isolated from BV2 cells exposed to LPS, and LPS + EFA for 24 h and untreated cells (three to four different cultures in triplicate for each treatment), and frozen retinas from control, untreated *rd10* and *rd10* + EFA mice at P18 (nine retinas for each group) using the NZY Total RNA Isolation Kit (#MB13402, Nzytech, Lisboa, Portugal), following the manufacturer’s protocol. RNA concentration was determined by spectrophotometry on the NanoDrop 2000 (Thermo Fisher Scientific, Wilmington, DE, USA). Then, cDNA was synthesized starting from 200 ng to 250 ng of RNA by reverse transcription using the PrimeScript™ RT Reagent Kit (Perfect Real Time) (#RR037A, Takara-Bio, Otsu, Japan), following the manufacturer’s instructions. The cycling conditions consisted of reverse transcription at 37 °C for 15 min and inactivation of reverse transcriptase at 85 °C for five seconds.

The relative expression of *Alox5*, *Alox15*, *Alox8*, *Elovl4*, *Tnfa*, *Tnfr1*, *Il1b*, *Il6*, *Il10*, *Il18*, *Arg1*, *Nos2* (inducible nitric oxide synthase), *Cd163* and *Gfap* was measured in BV2 and/or retinas by real-time PCR using a thermal cycler (LightCycler® 480 System; Roche, Basel, Switzerland). For TaqMan gene expression assay, the specific TaqMan probes were: Mm01182742_m1(*Alox5*), Mm00507789_m1 (*Alox15*), Mm01325281_m1 (*Alox8* homologue of human *ALOX15B*), Mm00521704_m1 (*Elovl4*), Mm00443260_g1 (*Tnfa*), Mm00441883_g1 (*Tnfr1*), Mm00434228_m1 (*Il1b*), Mm00446190_m1 (*Il6*), Mm01288386_m1 (*Il10*), Mm00434226_m1 (*Il18*), Mm00475988_m1 (*Arg1*), Mm00440502_m1 (*Nos2*), Mm00474091_m1 (*CD163*) and Mm01253033_m1 (*Gfap*). We used the Premix Ex Taq master mix for probe-based, real-time PCR (#RR390A, Takara-Bio, Otsu, Japan). The *β2-microglobulin* (*B2m*) gene (Mm00437762_m1) was used as the housekeeping gene. Real-time PCR was performed with one cycle of denaturation of 30 s at 95 °C, continued by 40 cycles of five seconds denaturation at 95 °C, 30 s annealing at 60 °C and one cycle of extension at 50 °C for 30 s. Normalized values of untreated BV2 cells or control mice were normalized to one to determine the changes in the gene expression due to the incubation with LPS and/or EFA supplement or in untreated *rd10* mice and EFA-treated *rd10* mice.

### 2.13. Flow Cytometry of Retinal Tissue

Mice were euthanized, and the retinas removed from the eyeballs. Retinae were placed in the RPMI medium and dissociated by gentle trituration (using pipette tips). Dissociated retinal cells were collected by centrifugation at 300× *g* for 5 min. Retinal cells were washed with PBS containing 1% FBS and stained with the following antibodies against the corresponding antigens: CD45 Alexa 488 (2 µg/mL, #103121, Biolegend, Amsterdam, The Netherlands), CD11b APC (5 µg/mL, #101211, Biolegend, Amsterdam, The Netherlands) and CD86 PE-Cy7 (1µg/mL, #105115, Biolegend, Amsterdam, the Netherlands) for 30 min at room temperature in the dark, as previously described [32]. After staining, the cells were centrifuged at 300 g for 5 min; the supernatant was discarded and the pellets resuspended in 200 µL of PBS with propidium iodide (PI) at 1 µg/mL. Both retinas comprised a single sample; thus, each sample represented the entire population of immune cells in two retinas. 

Flow cytometry of samples was performed using a CytoFLEX S (Beckman Coulter Life Sciences, Indianapolis, IN, USA), equipped with 4 lasers and 13 fluorescence detectors. Data acquisition was performed using the CytExpert 2.3 software (Beckman Coulter Life Sciences, Indianapolis, IN, USA). The gating strategy was defined to exclude the dead cells, defined as the cells stained with PI, and the aggregates, using the forward scatter area (FSC-A) and forward scatter height (FSC-H). After the selection of single and live cells, the double expression of CD45 and CD11b was used to define the resident microglia (CD45low/CD11b+). Selecting the CD45low/CD11b+ population, we quantified the concentration of these cells in our samples (events/µL) and their expression percentage of the CD86 marker (as %). Analysis was performed using at least seven mice (both retinas) for each experimental group. 

### 2.14. Enzyme-Linked Immunosorbent Assay of TNFα

TNFα concentration was quantified in the culture medium of BV2 cells exposed to LPS, and LPS + EFA for 24 h, or untreated cells, and in frozen retinas from control, untreated *rd10* and *rd10* + EFA mice at P18 using a highly sensitive ELISA kit from FineTest (#AQ-M0183, FineTest, Wuhan Fine Biotech Co., Ltd., Diaclone, Wuhan, China), according to manufacturer’s instructions. Readings were performed at 450 nm in a microtiter plate reader Halo LED 96 (Dynamica Scientific Ltd., Livingston, UK). TNFα concentration was expressed as pg/mL or pg/mg protein. Four different cultures were used per treatment, with three replicas for each treatment. Eight animals/groups were used.

### 2.15. Determination of Redox Status

Extracellular nitrites (stable end-product of nitric oxide (NO) and nitrate determination (NOX): NOX was measured in a culture medium of BV2 cells by spectrophotometric GRIESS reaction using nitrate reductase [33]. NOX levels were expressed as nmol/mL. Retinal redox status was evaluated by measuring the activities of catalase (CAT), superoxide dismutase (SOD), total antioxidant capacity (TAC), formation of thiobarbituric acid reactive substances (TBARS, indicator of lipid peroxidation) and the content of protein carbonyl adducts (CAR) in retinal tissue from at least seven retinas for each experimental groups. Retinas were homogenized in 5 mM phosphate buffer pH 7, 0.9% NaCl, 0.1% glucose, and centrifuged at 10,000× *g* for 15 min at 4 °C as previously described [30]. 

TAC determination: TAC was measured by detecting the oxidation of 2,2′-azino-di [3—ethylbenzthiazoline sulphonate] (ABTS) by metmyoglobin (absorbance at 405 nm) (#709001, Cayman Chemical, Ann Arbor, MI, USA). TAC levels were expressed as nmol of antioxidants/mg protein. 

Determination of SOD activity: This was based on the dismutation of superoxide oxygen and hydrogen peroxide with a commercial kit. A tetrazolium salt reacted with superoxide radicals generated by xanthine oxidase and hypoxanthine and the formazan dye was colorimetrically measured (absorbance al 450 nm) (#706002, Cayman Chemical, Ann Arbor, MI, USA). Total SOD activity was expressed as U/mg protein. 

Determination of CAT activity: This was based on the reaction of catalase with methanol in the presence of H_2_O_2_. The formaldehyde produced was measured colorimetrically with 4-amino-3-hydrazino-5-mercapto-1,2,4-triazole (absorbance at 540 nm) (#707002, Cayman Chemical, Ann Arbor, MI). CAT activity was expressed as nmol of formaldehyde/min.mg protein. 

Lipid peroxidation: This was evaluated by measuring the formation of TBARS (including malondialdehyde, MDA), which were formed as a byproduct of lipid peroxidation (#10009055, Cayman Chemical, Ann Arbor, MI). TBARS levels were expressed as nmol of MDA/mg protein. 

Protein carbonylation: CAR were measured using fluorescein-5-thiosemicarbazide (FTC), a fluorescent probe that covalently reacted with oxidized residues on proteins. FTC generated a stable fluorometric signal that was monitored (Ex/Em 485/535 nm) (#ab235631, Abcam, Cambridge, UK). CAR content was expressed as nmol/mg protein.

Protein concentration: This was measured using the bicinchoninic acid (BCA) protein assay (#23225, BCA Kit; Pierce Scientific, CA, USA). Protein content was expressed as mg/mL.

### 2.16. Statistical Analysis

Statistical analyses were performed using GraphPad Software 9.0 (Prism; GraphPad Software, Inc., San Diego, CA, USA). The normal distribution of data was analyzed by Shapiro–Wilk and Kolmogorov–Smirnov tests. For in vitro studies, comparisons between control, LPS and LPS + EFA groups were performed using one-way ANOVA followed by Dunnett’s, Tukey’s or Holm–Sídák’s multiple comparisons tests or unpaired *t*-test. For in vivo studies, comparisons between control, *rd10* and *rd10* + EFA groups were performed using the Kruskal-Wallis test followed by Dunn’s multiple comparisons test or one-way ANOVA followed by Tukey’s multiple comparisons test depending on data distribution (non-parametric analysis or parametric). In some cases, comparisons between *rd10* and *rd10* + EFA were performed using the Mann–Whitney U test. A *p*-value < 0.05 was considered statistically significant. The data were plotted using the Graph Pad Software 9.0. The data were presented as the mean ± SEM.

## 3. Results

### 3.1. ALOX5 and ELOVL4 Relevance in the RP Mechanistic Map Obtained by the ML Model

We evaluated the overall impact of target genes *Alox5* and *Elovl4*, both related to the metabolism of fatty acids and lipids, including SPMs, over the built RP map that modelled signaling activity under normal conditions. After the machine learning model computed global and SHAP relevancies, we concluded that both genes were highly relevant for the model, obtaining a relevance value of 3.60 × 10^−3^ for ELOVL4 and of 3.41 × 10^−3^ for ALOX5 (62nd and 65th in the ranking out of 125, respectively).

Indeed, when we evaluated the drugs targeting *Alox5* and *Elovl4*, we found that, according to the DrugBank database, most of the drugs have an inhibitor or antagonist effect, while diclofenac, omega-3 fatty acids, omega-6 fatty acids, isocapent, Vayarin and omega-3-carboxylic acids, act as substrate or potentiator of the activity of ALOX5 and/or ELOVL4 (Table S1, Supplementary Materials).

We wanted to evaluate the impact of *Alox5* and *Elovl4* genes over the functional landscape of the disease; therefore, we disaggregated the individual contribution of each target and each circuit to the prediction model, obtaining the impact of ALOX5 and ELOVL4 over all the circuits involved in RP, according to ORPHANET and KEGG. The results are listed in Tables S2 and S3 (Supplementary Materials). The SHAP results showed that ALOX5 was relevant to 18 circuits within 7 signaling pathways, while ELOVL4 appeared as a relevant target for 81 circuits belonging to 20 pathways; of those, 7 circuits were associated with both ALOX5 and ELOVL4 targets, related to Ras signaling pathway, PI3K-Akt signaling pathway, natural-killer-cell-mediated cytotoxicity and regulation of actin cytoskeleton, and most of them were mediated by FAS. After the functional annotation of all the effector genes of these relevant circuits, we found that functions were mainly linked to actin binding, neurogenesis, transcription regulation, cell cycle, cell adhesion, apoptosis and, interestingly, with fatty-acid and lipid metabolism.

Once we assessed the putative impact of ALOX5 and ELOVL4 in RP, we analyzed them together with other ALOXs in *rd10* mice.

### 3.2. Altered Temporal Profile of Alox5, Alox8, Alox1, and Elovl4 in the Retinas of rd10 Mice

**Figure 3:**
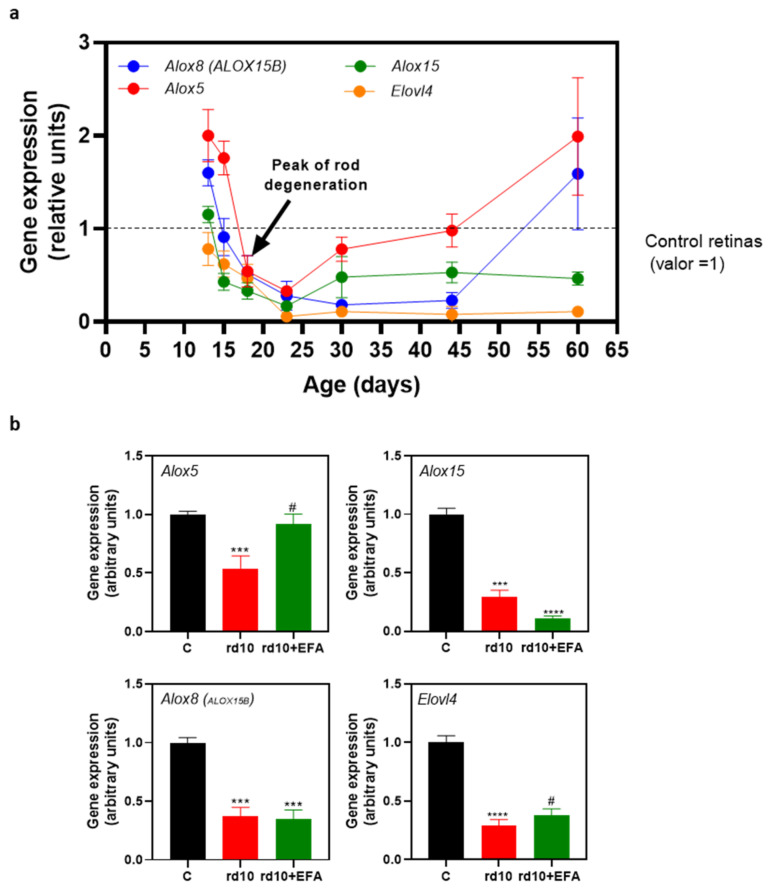
Genes involved in SPM synthesis were downregulated in *rd10* mice. Temporal profile of gene expression in retinas from *rd10* mice and age-matched control retinas (**a**); Effect of oral administration of EFA supplement, a concentrate of omega-3 fatty acids and SPM precursors, in gene expression of these enzymes at P18 (**b**). The *rd10* mice were treated with EPA supplement (2.82 mg/body weight) daily from P9 to P18. ANOVA followed by Tukey’s multiple comparisons test (*Alox5* gene) or Kruskal-Wallis test followed by Dunn’s multiple comparisons test (*Alox15*, *Alox8* and *Elovl4* genes) was used to compare three groups and Mann–Whitney U test was used to compare *rd10* vs. *rd10* + EFA. *** *p* < 0.001; **** *p* < 0.0001 for differences between control and *rd10* mice or *rd10* + EFA mice; ^#^ *p* < 0.05 for differences between *rd10* and *rd10* + EFA mice. Data were presented as mean ± standard error of the mean (SEM). We analyzed ten mice for each group. *Alox:* lipoxygenase gene; EFA: essential fatty acid.

We analyzed the genes of the rate-limiting enzymes responsible for the generation of SPMs (*Alox5, Alox8, and Alox15*) or elovanoids (*Elovl4)* in *rd10* mouse retinas compared to age-matched control retinas from P13 to P60 (Figure 3a). We observed a significant upregulation of the *Alox5* gene (*p* < 0.005, unpaired *t*-test) and to a lesser extent, the *Alox8* gene, preceding the first peak in rod degeneration (P18) (Table 3) [30].

*Elovl4* expression was downregulated (*p* < 0.05, unpaired *t*-test) throughout retinal degeneration. From P18 to P44, there was a persistent downregulation of *Alox15,* and *Alox8* genes (*p* < 0.05, unpaired *t*-test). *Alox5* expression was very low at P23 (*p* < 0.0001, unpaired *t*-test) and then returned to the control values during the later stages of RP degeneration (P44–P60) (Figure 3a and Table 3). *Alox8* expression also returned to the control values at P60. Therefore, these findings suggested that RP progression, at least in *rd10* mice, is accompanied by an imbalance in the expression of the key enzymes involved in SPM and elovanoid synthesis.

### 3.3. SPM Precursors Shifted M1 Microglia to M2 in LPS-Treated BV2 Cells

**Figure 4:**
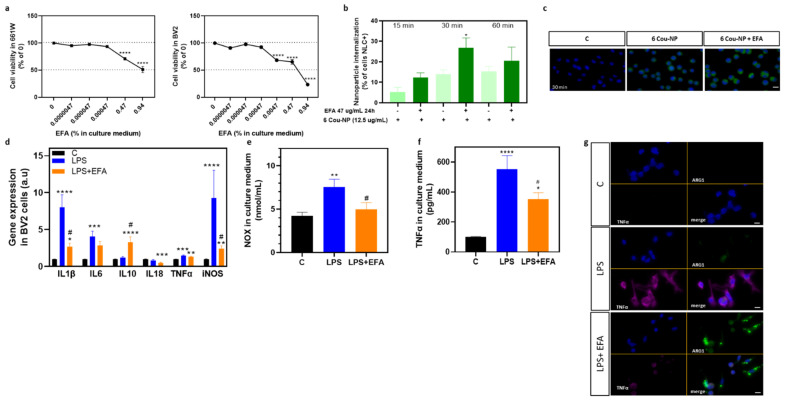
SPM precursors diminished inflammatory markers and switched microglia phenotype from M1 (pro-inflammatory) toward M2 (pro-resolving) in LPS-treated BV2 cells. EFA supplement dose–response curve in 661W and BV2 cells incubated to different concentrations of EFA supplement for 24 h (MTT assay) (**a**); Internalization of nanostructured lipid carriers (NLCs) loaded with coumarin 6 in BV2 cells exposed to EFA supplement by flow cytometry (**b**) and microscopy (**c**), scale bar: 10 µm; Effect of EFA supplement in LPS-mediated cytokine and *Nos2* gene expression (**d**), nitrite (NOX) formation (**e**), and TNFα release (**f**) in BV2 cells; Immunofluorescence images showing localization of TNFα and ARG1 in BV2 cells treated with LPS with or without EFA supplement (**g**), scale bar: 10 µm. BV2 cells were pretreated with or without 47 µg/mL EFA supplement for 2 h. Then, cells were exposed to 1 µg/mL LPS for 24 h. Results were expressed as mean ± SEM of six (**a**), four to five (**b**–**e**) and four independent (**f**,**g**) experiments in triplicate. Ordinary one-way ANOVA followed by Dunnett’s multiple comparisons test (**a**), by Tukey’s multiple comparisons test (**d**,**e**), Kruskal-Wallis test followed by Dunn’s multiple comparisons test (**f**) and unpaired *t*-test (**b**). * *p* < 0.05, ** *p* < 0.01, *** *p* < 0.001 and **** *p* < 0.0001 vs. C (or xero) and ^#^ *p* < 0.05 LPS alone vs. LPS + EFA. C, control; LPS, lipopolysaccharide; EFA, essential fatty acid; NOX, nitrites.

To test the effects of the EFA supplement on neuroinflammation, we first examined whether it was toxic to the retinal and microglia cells, 661W and BV2, respectively. We diluted the EFA supplement in the culture medium from 0.0000047 to 0.94 mg/mL. After 24 h, the EFA supplement did not exhibit cytotoxicity at lower doses; however, at the highest doses, 0.47 mg/mL and 0.94 mg/mL, cytotoxicity was experienced by the 661W and BV2 cells (Figure 4a). BV2 cells were apparently more sensitive to the EFA supplement than 661W cells. We assessed whether the EFA supplement promoted internalization of 6Cou-NP in BV2 cells. We observed that the EFA supplement significantly increased the number of NLC-positive cells after 30 min compared (*p* < 0.05, unpaired *t*-test) with untreated cells (Figure 4b,c).

As has been previously described, reactive polarized microglia express different cytokines and cell surface markers. To evaluate the effect of the EFA supplement on M1/M2 polarization, we analyzed the expression or content of M1 (*Il1b, Il6, Tnfa, Nos2*, NOX) and M2 (*Il10* and ARG1) markers in BV2 cells treated with 47 µg/mL of the EFA supplement for two hours, followed by LPS (1 μg/mL) for 24 h. LPS induced alterations in the expression of M1 markers (Figure 4b). Following LPS treatment, *Il1b*, *Il6*, *Tnfa*, *Nos2* expression (Figure 4d), as well as extracellular NOX and TNFα concentrations, significantly increased (*p* < 0.05 ANOVA, Tukey’s test or Holm–Sídák’s test) compared with unstimulated cells (Figure 4e,f).

The EFA supplement suppressed, at least partly, LPS-induced M1 polarization and promoted M2 polarization in BV2 cells. SPM precursors significantly reduced the gene expression of LPS-induced inflammatory molecules *Il1b* and *Nos2* (*p* < 0.05 ANOVA, Tukey’s test) (Figure 4d). The reduction in NOX (*p* < 0.05 ANOVA, Tukey’s test) found in the culture medium supported *Nos2* downregulation (Figure 4d). The EFA supplement appeared to reduce the content of pro-inflammatory genes *Il6* and *Tnfa,* and TNFα (Figure 4b,d,e). Moreover, SPM precursors significantly increased gene expression of the anti-inflammatory gene and marker of M2 phenotype, *Il10*. Immunofluorescence of ARG1 also suggested that the EFA supplement shifted the microglia phenotype from M1 toward M2 in LPS-treated BV2 cells (Figure 4g).

### 3.4. Oral Administration of SPM Precursors Ameliorated Retinal Dysfunction and Reduced PR Degeneration in rd10 Mice

Once we confirmed the imbalance in gene expression in *Alox5, 8, 15* and *Elovl4* in *rd10* mouse retinas and analyzed the action of the EFA supplement in BV2 cells, we examined the effect of the EFA supplement orally administered to *rd10* mice. Firstly, we analyzed whether SPM precursors affected the gene expression of the enzymes involved in SPM and elovanoid synthesis. As is shown in Figure 3b, the EFA supplement normalized the gene expression of *Alox5* (*p* < 0.05, one-way ANOVA, Tukey’s test) without affecting the other enzymes *Alox8, Alox15* and *Elovl4*. 

#### 3.4.1. ERG Recordings

As has been previously described, chronic inflammation is observed during RP progression and it could exacerbate retinal degeneration. We assessed whether SPM precursors delayed retinal degeneration at P18, when we observed significant retinal dysfunction and a peak in PR degeneration [26,29,33].

**Figure 5:**
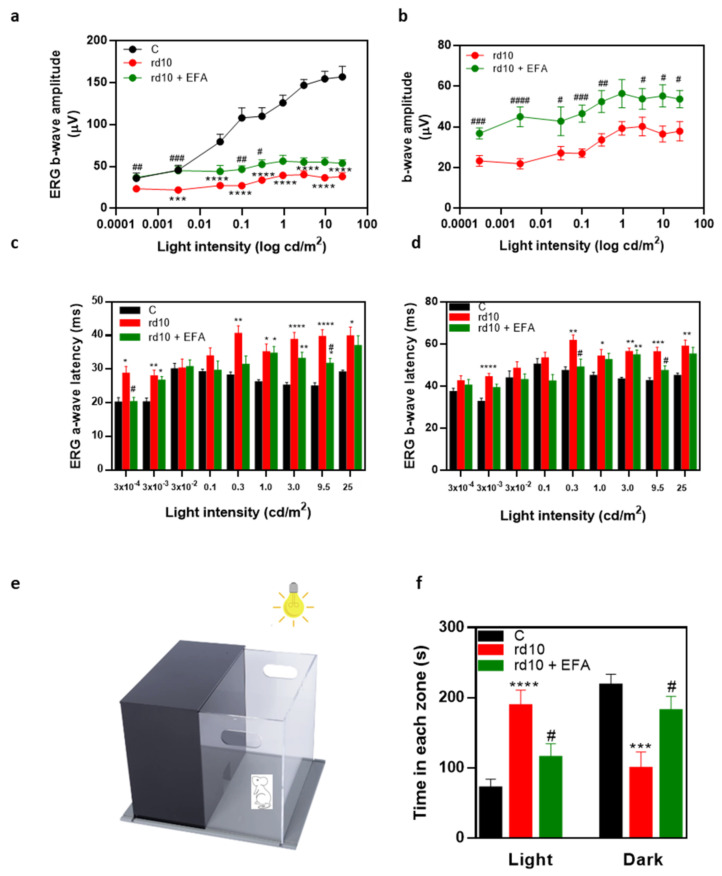
SPM precursors improved visual function in *rd10* mice at P18. Amplitudes of ERG b-wave (**a**,**b**); a- and b- wave implicit time or latencies recorded (**c**,**d**) from dark-adapted control mice, C, untreated *rd10* mice and EFA-treated *rd10* mice (*rd10* + EFA) at different intensities of light stimuli; light/box test for these three groups (**e**,**f**). The *rd10* mice were treated with EFA supplement daily from P9 to P18. Kruskal-Wallis test followed by Dunn’s multiple comparisons test or ANOVA followed by Tukey’s multiple comparisons tests were used to compare three groups and Mann-Whitney test was used to compare *rd10* vs. *rd10* + EFA. * *p* < 0.05; ** *p* < 0.01; *** *p* < 0.001; **** *p* < 0.0001 for differences between control and *rd10* mice or *rd10* + EFAmice; ^#^ *p* < 0.05; ^##^ *p* < 0.01; ^###^ *p* < 0.005, ^####^ *p* < 0.0001 for differences between *rd10* and *rd10* + EFA mice. Data were presented as mean ± standard error of the mean (SEM). We analyzed at least ten mice for each group.

We examined retinal function by measuring the electrical activity of the retina in response to different light stimuli at P18. At this age, as has been previously described [27], global ERG responses were considerably reduced in untreated *rd10* mice (Figure 5a,b). We did not observe significant differences between males and females in this study (data not shown). Under scotopic conditions, there was a significant decline in b-wave amplitudes at most flash intensities compared to the control mice (*p* < 0.0001, Kruskal-Wallis, Dunn’s test) (Figure 5a). The a-wave was barely detectable. EFA-treated *rd10* mice showed better ERG recordings than untreated *rd10* mice. As is shown in Figure 5b, most scotopic b-wave amplitudes were significantly higher in EPA-treated *rd10* mice than in untreated *rd10* mice (*p* < 0.05, Mann–Whitney test). The implicit time of a- and b-waves of untreated *rd10* mice (approximately 28–40 ms and 42–60 ms, respectively) were significant longer than of control mice (approximately 20–29 ms and 32–50 ms) for multiple light intensities (*p* < 0.05, Kruskal-Wallis, Dunn’s test) (Figure 5c,d). The EFA supplement had a less pronounced effect in reducing a- and b-wave implicit times (Figure 5c,d). Therefore, oral administration of the EFA supplement partially restored retinal function in *rd10* mice at P18. 

#### 3.4.2. Light Aversion

In order to evaluate function, we examined the behavior of the mice in an illuminated open field that contained a dark zone (light/dark box) (Figure 5e). We placed the mice in the illuminated zone and recorded their movements for a total of 300 s including the time spent in each zone, and the number of entries in each zone. Mice have an innate aversion to brightly illuminated areas and they prefer dark environments. In Figure 5f, we showed the time that the mice spent in the light and dark zones. Control mice spent less than 100 s in the light zone. However, *rd10* mice spent more time in the light zone (~200 s) than control mice (*p* < 0.0001, one-way ANOVA, Tukey’s test). After EFA supplementation, the time that *rd10* mice spent in the light zone significantly decreased (~100 s) and the time spent in the dark zone increased compared with the untreated *rd10* mice (*p* < 0.05, one-way ANOVA, Tukey’s test). At P18, the control mice had good light perception, and they preferred the dark zone. However, *rd10* mice showed poor light perception, but the EFA supplementation partly restored their light perception. These results supported ERG studies and suggested the EFA supplement improved the visual function of *rd10* mice.

#### 3.4.3. PR Degeneration

**Figure 6:**
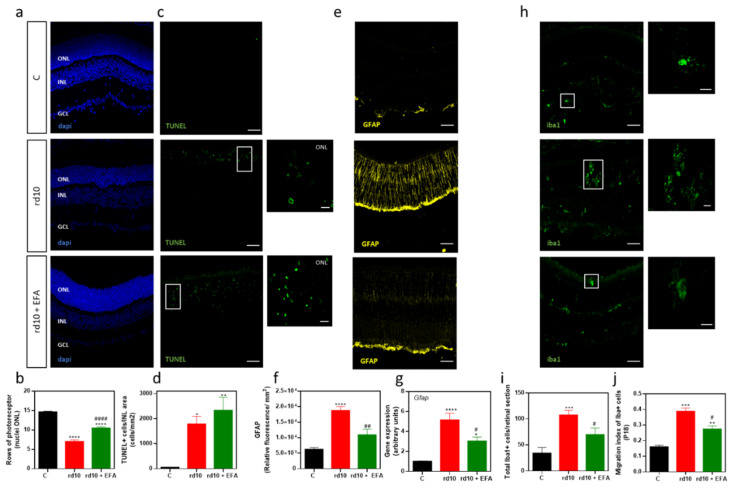
SPM precursors decreased retinal degeneration and microglia activation in *rd10* mice at P18. Representative photomicrographs of retinal sections showing DAPI staining (**a**) and quantification of number of rows of nuclei at ONL (**b**); Representative photomicrographs of retinal sections showing TUNEL-positive cells in ONL (**c**) and quantification of the number of TUNEL-positive nuclei cells (**d**); Representative photomicrographs of retinal sections showing GFAP labelling (**e**) and its relative fluorescence (**f**); Gene expression of GFAP in retinal homogenates (**g**); Representative photomicrographs of retinal sections showing Iba1-positive cells (**h**) and quantification of Iba1-positive cells (**i**) and migration index of microglia cells (**j**) from control mice (C), untreated *rd10* mice and EFA-treated *rd10* mice (*rd10* + EFA). Higher magnification images (scale: 10 µm) showing microglial process infiltration in ONL and phagosome formation in *rd10* mouse retinas. scale: 10 or 50 µm. *rd10* mice were daily treated with EFA supplement from P9 to P18. Then, eyes were processed for histological analysis or gene expression. ONL: outer nuclear layer; INL: inner nuclear layer; GCL: ganglion cell layer. One-way ANOVA followed by Tukey’s multiple comparisons test, * *p* < 0.05; ** *p* < 0.01; *** *p* < 0.001; **** *p* < 0.0001 for differences between control and *rd10* mice or *rd10* + EFA mice; ^#^ *p* < 0.05; ^##^ *p* < 0.01; ^####^ *p* < 0.0001 for differences between *rd10* and *rd10* + EFA mice. Data were presented as mean ± standard error of the mean (SEM). We analyzed at least nine mice for each group.

We assessed whether the EFA supplement prevented PR cell death by histological evaluation. We quantified the number of the remaining PRs (number of rows of nuclei at ONL), and the number of TUNEL-positive cells (DNA fragmentation of dying cells) (Figure 6a–d). At P18, EFA supplementation significantly reduced cell loss at ONL in *rd10* mice compared to untreated *rd10* mice (*p* < 0.0001, ANOVA, Tukey’s test) (Figure 6a,b). Results of the TUNEL assay suggested a slight, but not significant increase in the number of TUNEL-positive cells at the ONL in EFA-treated compared to untreated *rd10* mice (Figure 6c,d).

### 3.5. Oral Administration of SPM Precursors Reduced Reactive Gliosis, Microglia Migration, and M1-Microglia Markers and Increases M2-Microglia Markers in rd10 Mouse Retinas at P18

As we previously described, RP progression is accompanied by a sustained and chronic inflammation including reactive gliosis, microglia activation and migration, and upregulation of pro-inflammatory molecules such as TNFα, IL6 or IL1β [26,33,34]. We analyzed whether EFA supplementation was capable of reducing this inflammation in *rd10* mice at P18.

#### 3.5.1. Reactive Gliosis and Iba1-Positive Cell Migration

We observed that SPM precursors reduced the increase in GFAP, a type III intermediate filament protein, which is a known sensitive marker for retinal gliosis, in *rd10* mouse retinas (*p* < 0.0001, ANOVA, Tukey’s test) (Figure 6e,f). In addition, we confirmed its decrease, analyzing the gene expression in retinal homogenates (Figure 6g).

During RP progression, activated microglia (mainly M1 phenotype) migrate toward degenerated PRs changing their morphology to amoeboid because of their indiscriminate phagocytic activity [34]. We labeled microglia cells with anti-Iba1 and quantified the number of Iba1-positive cells and microglial migration from the inner to the outer retina, as previously described [35] (Figure 6h–j). In Table 4, we showed the percentage of Iba1-positive cells in each nuclear or plexiform layer. For the control mice, the microglia were mainly limited to the inner retina layers (IPL and GCL) with a horizontally ramified shape. For the untreated *rd10* mice, the microglia migrated, infiltrated the ONL via radially oriented cellular projections, and intercalated closely with the PR nuclei, as has been previously described by Zhao et al. [34]. Moreover, these microglia acquired a rounded and amoeboid morphology (Figure 6h). The EFA supplement reduced the number of Iba1-positive cells, microglial migration and infiltration to ONL, where degenerating PRs are located (*p* < 0.05, ANOVA, Tukey’s test), as well as changing its morphology (Figure 6h). Microglia of EFA-treated *rd10* mice showed a more ramified morphology than those from untreated *rd10* mice. 

#### 3.5.2. M1 and M2 Microglia Markers

**Figure 7:**
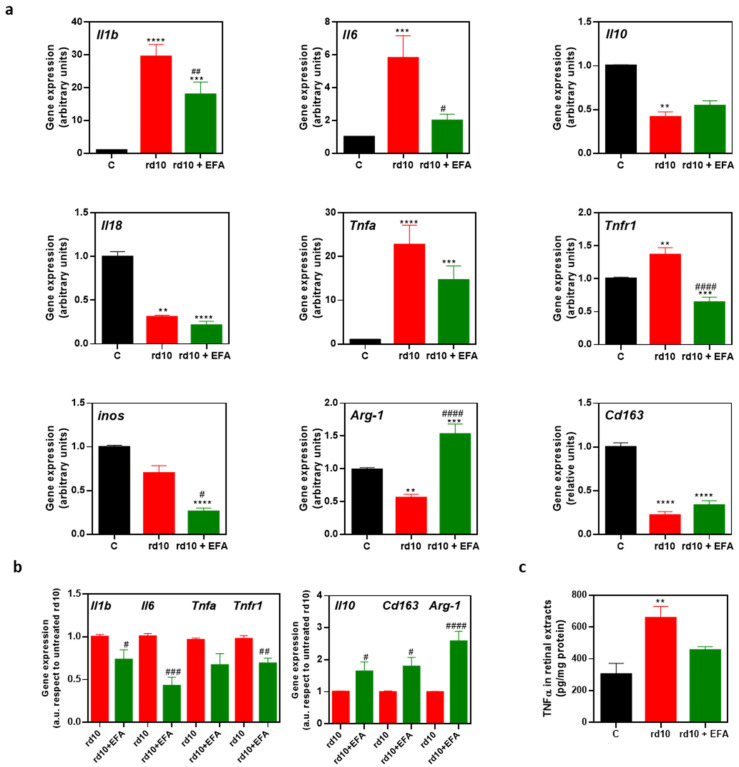
SPM precursors restored M2 pro-resolving phenotype in *rd10* mouse retinas at P18. Expression of several M1- and M2-phenotype genes in untreated *rd10* mice and EFA-treated *rd10* mice (*rd10* + EFA) compared to control mice (C) (**a**); or compared to untreated *rd10* mice (**b**); TNFα content in retinal extracts (**c**). *rd10* mice were treated daily with EFA supplementation from P9 to P18. RNA was extracted from frozen retinas and real-time quantitative PCR was performed to evaluate gene expression. One-way ANOVA or Kruskal-Wallis test followed by Tukey’s or Dunn’s multiple comparisons test was carried out for (**a**,**c**), unpaired *t*-test was carried out for (**b**), ** *p* < 0.01; *** *p* < 0.001; **** *p* < 0.0001 for differences between control and *rd10* mice or *rd10* + EFA mice; ^#^ *p* < 0.05; ^##^ *p* < 0.01; ^###^ *p* < 0.001; ^####^ *p* < 0.0001 for differences between *rd10* and *rd10* + EFA mice. Data were presented as mean ± standard error of the mean (SEM). We analyzed at least ten mice for each group.

We then explored whether oral administration of the EFA supplement could modify microglia phenotypes (M1 and M2), polarizing microglia toward a pro-resolving state (M2). We analyzed the gene expression of M1 (*Il1b, Il6, Il18, Tnfa, and Nos2*) and M2 (*Arg1, Il10 and Cd163*) markers in retinal homogenates. As us shown in Figure 7a, at P18, we observed an upregulation of the M1 genes *Il1b, Il6,* and *Tnfa* and a downregulation of the M2 markers *Il10, Arg1* and *Cd163* in *rd10* mice compared to control mice (*p* < 0.01, ANOVA or Kruskal-Wallis test). *Tnfa* upregulation occurred along with the upregulation of its receptor *Tnfr1*. However, we showed a downregulation of the M1 markers *Nos2* and *Il18* (Figure 7a). EFA supplementation significantly reduced *Il1b, Il6, Tnfr1* and to a lesser extent, *Tnfa* gene expression and TNFα content (Figure 7a,c). EFA supplementation significantly upregulated *Arg1* compared to untreated *rd10* and control mice (Figure 7a).

The data suggested that SPM precursors induced a slight increase in *Il10* and *Cd163* expression in *rd10* mice compared to untreated *rd10* mice. However, the expression of these genes was below the normalized control value (Figure 7a). To further interrogate the action of EFA supplementation on M1/M2 markers, we normalized values from untreated *rd10* mice and compared them with EFA-treated *rd10* mice (Figure 7b). EFA supplementation significantly reduced M1 markers and increased M2 markers (*p* < 0.05, unpaired *t*-test), suggesting a polarization toward M2, a more pro-resolving phenotype.

**Figure 8:**
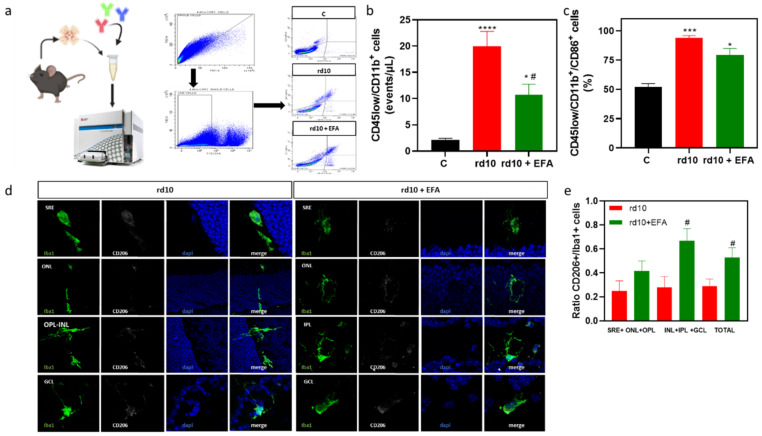
SPM precursors restored pro-resolving microglia by reduction in CD86 and increased CD206 markers in *rd10* mouse retinas at P18. Scheme of the procedure for the identification of CD45^low^/CD11b^+^ cells by flow cytometry and representative dot plots from control, C, untreated *rd10* and EFA-treated *rd10* mice (*rd10* + EFA) (**a**). Selection of single cells using the FSC-A and FSC-H signals. Propidium iodide (PI) signal was used to discard the dead cells (PI^+^) on the single-cell gated cells. The double selection discarding aggregates and dead cells were applied to the CD45^−^FITC/CD11b^−^APC dot plot. Quantification of CD45^low^/CD11b^+^, (microglia) (**b**) and CD45^low^/CD11b^+^ expressing CD86 on their surface (**c**); Representative photomicrographs of retinal sections showing double labelling for Iba1 and CD206-positive cells (**d**) and the ratio of CD206^+^/Iba1-positive cells in the inner and outer retina (**e**). One-way ANOVA or Kruskal-Wallis test followed by Tukey’s for (**b**) or Dunn’s multiple comparisons test was used for (**c**) and unpaired *t*-test was used for (**e**), * *p* < 0.05; *** *p* < 0.001; **** *p* < 0.0001 for differences between control and *rd10* mice or *rd10* + EFA mice; ^#^ *p* < 0.05 for differences between *rd10* and *rd10* + EFA mice. Data were presented as mean ± standard error of the mean (SEM). We analyzed at least eight mice for each group.

Analysis of gene expression suggested that SPM precursors shifted microglia cells from the M1 phenotype to the M2 phenotype at P18. Therefore, we explored this M1/M2 polarization, and we analyzed microglia populations (CD45^low^/CD11b^+^), specifically which percentage of these cells were CD86-positive cells (M1 marker) in freshly suspended cells of whole retinae (Figure 8a). The results demonstrated that *rd10* mice had more CD45^low^/CD11b^+^ cells than control mice (*p* < 0.0001, ANOVA, Tukey’s test) suggesting microglia proliferation (Figure 8b). EFA supplementation significantly reduced these populations (CD45^low^/CD11b^+^) (*p* < 0.05, ANOVA, Tukey’s test). CD86-positive microglia cells were dominant in *rd10* mice and even in EFA-treated *rd10* mice (*p* < 0.05, Kruskal-Wallis, Dunn’s test) (Figure 8c). However, we observed a tendency of EFA supplementation to reduce the surface expression of CD86 (Figure 8c).

Finally, we analyzed whether EFA supplementation increased CD206 (M2 marker) in retinal cryosections. Our findings suggested that administration of the EFA supplement increased the ratio of CD206^+^/Iba-positive cells in *rd10* mouse retinas (Figure 8d,e). We observed that the increase in CD206-positive cells was more evident and significant in the inner retina than in the outer retina in EFA-treated retinas (*p* < 0.05, unpaired *t*-test) (Figure 8e).

### 3.6. Oral Administration of SPM Precursors Ameliorated Oxidative Stress in rd10 Mouse Retinas at P18

Inflammation and oxidative stress are interrelated, and both contribute to the pathogenesis of RP. As has been previously described, *rd10* mouse retinas had an imbalanced redox status with lower antioxidant response and higher levels of oxidative stress markers [27]. We studied whether EFA supplementation had any effect on redox status in *rd10* mice at P18. We measured total SOD and CAT activities and TBARS in retinal homogenates. As is shown in Table 5, we observed that EFA supplementation significantly reduced oxidative stress markers TBARS and CAR (*p* < 0.05 and *p* < 0.01, Kruskal-Wallis, Dunn’s test). However, EFA supplementation did not significantly affect the activity of the antioxidant enzymes of *rd10* mouse retinas.

## 4. Discussion

Inherited retinal dystrophies (IRD) such as RP are genetic diseases. However, they have an important inflammatory component exacerbating their progression. In particular, RP progression is accompanied by chronic and sustained inflammation. Previous studies have suggested that lipid mediators, such as SPMs, derived from ω-6 and ω-3 LC-PUFAs (DHA, EPA) could regulate retinal inflammation in different retinopathies such as diabetic retinopathy (DR) or age-related macular degeneration (AMD) [36,37].

In the current study, we assessed the putative involvement of SPMs in the pathogenesis and neuroprotection of RP. Firstly, we analyzed whether some of the main enzymes involved in SPM synthesis were altered in a model of RP, the *rd10* mouse. Secondly, we assessed whether oral administration of an EFA supplement could ameliorate retinal degeneration by modulating inflammation, especially the microglia phenotype, a key cell in immune system. We also evaluated the in vitro actions of an EFA supplement in BV2 cells, a cell line of microglia.

In this study, we used mechanistic maps for selecting *Elovl4* and *Alox5* genes, both of which are involved in SPM and elovanoid synthesis. These maps have been proven to be useful tools to simulate and predict cellular events, both under normal condition or in association with diseases. Thus, modeling cell activity allows us to evaluate the potential effect that certain interventions, such as knocking down genes or administering drugs with known mechanisms of action, would have over the map or the functional landscape of a given disease, in this case, RP. The HiPathia algorithm, which uses gene expression activities along with signaling map topology to simulate cellular processes, has been successfully proven to predict cell activities related to cancer hallmarks [20,38] as well as the effect of protein inhibitions on cell survival [39]. The model we used in this study improved upon the COVID-19 drug repurposing tool proposed by the authors in [21], where an ill-conditioned circuit could drag the model performance and stability. We also functionally annotated the relevant circuits or sub-pathways obtained by the ML model using UniProt KB and Gene Ontology BP annotations and manually curated the associated functions. In our case, these maps predicted that ALOX5 and ELOVL4 could be important for RP progression.

We observed a visible upregulation of ALOXs expression before retinal degeneration at P13–P15 [30]. At P18, ALOXs expression dropped significantly along with survival of the PR cells. This downregulation continued until P30. *Alox5* and *Alox8* (homologues of human *ALOX15B*) returned to normal values but, *Alox15* remained very low during the later stages of RP (P44–P60). On the other hand, *Elovl4* expression was dramatically downregulated throughout RP progression (from P15 to P60). These findings suggested that synthesis of SPMs could be altered, affecting their pro-resolving activities. As is shown, the temporal profiles of *Alox5, 8,* and *15* and *Elovl4* were different. In macrophages, the resolution phase is linked to a strong upregulation of ALOX15 and synthesis of LX, RV, PD and MaR that facilitate the resolution of inflammation [40]. In human macrophages, ALOX15 has an expression that is Th2 cytokine dependent (IL4 and IL3) but ALOX15B (Alox8 in mice) is constitutively expressed, although it can be further induced by a pro-inflammatory stimuli [41]. Both catalyze the peroxidation of LC-PUFAs to the same derivatives [42]. We speculate that the different expression of *Alox8* and *Alox15* was due to the retinal inflammatory status at each stage of the disease.

We previously observed that the pro-inflammatory genes *Tnfa* and *Il6* were upregulated before and during the peak in PR loss (P13–18). After that period, these inflammatory mediators decreased to the levels of the control values (P20–23). At P44, *Tnfa* slightly increased [30]. Overexpression of *Alox5* and *Alox8* prior to the onset of RP could have a detrimental effect due to excess LXs or promotion of lipid peroxidation [43]. For instance, in some models of retinal damage such as DR, *Alox5* upregulation was associated with oxidative damage [44]. In addition to ALOX5, ALOX12/15 inhibitors reported a reduction in free radicals, oxidative damage and cell death [45,46]. However, as was mentioned by Wang et al. (2020) more studies are needed to determine whether ALOX inhibition or redox homeostasis is responsible for the biological result in these situations [47]. It is also important to study the protein content of ALOXs and their localization because they can promote the synthesis of LXs or SPMs depending on their localization (e.g., nuclear vs. cytoplasmic ALOX5) [48]. Therefore, more studies are needed to clarify all of these issues.

Previous studies have shown that metabolites of ω-3 LC-PUFA through ALOX pathways contain anti-inflammatory molecules such as RVs that reduce ROS damage, inflammation and cell death in models of AMD and DR [49]. In *rd1* mice, lipoxin 4 (LXA4) biosynthesis through ALOX5 and ALOX15 and its receptor ALX/FPR2 were downregulated during the later stages of RP [50]. Thus, intravitreal injection of LXA4 delayed visual loss by inhibiting microglia activation and PR cell death. Apart from retinal diseases, some studies have indicated that inadequate levels of SPMs have been associated with sustained inflammation in neurodegenerative diseases such as Alzheimer’s disease or multiple sclerosis [51,52,53,54,55].

We found that the EFA supplement was biocompatible and had functional activity in retinal and microglia cells (661W and BV2 cell lines, respectively), corroborating recent findings in other cell types [19]. We observed a negative effect on cell survival when the EFA supplement concentration was higher than 47 µg/mL in the culture medium. In the presence of LPS, which stimulates the M1 phenotype in BV2 cells, EFA supplementation was capable of reducing the M1 markers *Il1b*, *Nos2* or *Tnfa* and promoting the M2 markers *Il10* and ARG1 in BV2 cells. Stimulation of phagocytes to clear/neutralize pathogens or the removal of dead cells by enhancing phagocytosis is one of the pro-resolving actions of SPMs [56]. In this sense, we observed that EFA supplementation enhanced the internalization of nanoparticles in BV2 cells.

Our findings suggested that daily oral administration of the EFA supplement normalized *Alox5* expression in *rd10* mice at P18. Simon et al. showed that a diet enriched in ω3 LC-PUFAs (DHA and EPA) and with low linoleic acid content upregulated retinal gene expression of *Alox5*, among others, in rats [57]. On the other hand, during *Alox5* upregulation (P13–P15), EFA supplementation did not appear to have a detrimental effect on cell survival.

We showed that EFA supplementation ameliorated retinal dysfunction and inflammation in RP. Several studies have been carried out to evaluate the ocular benefits of DHA or EPA supplementation in different retinopathies. In 2020, Schwartz et al. reviewed the available evidence on the beneficial effects of DHA or vitamin A in ameliorating RP progression. After analyzing four clinical trials, they concluded that evidence for the benefit of DHA or vitamin A was low. More studies with other outcome measures such as ERG recordings should be included [58]. In a previous systematic review other authors found no evidence for a beneficial effect of DHA in RP [59]. High dietary intake of ω3 LC-PUFAs was associated with a lower risk of developing AMD and protection against the progression of AMD [60,61]. For DR, DHA seems to be the most effective lipid to decrease free radicals, increase antioxidant response and prevent angiogenesis [62]. Dietary supplementation with a high-dose DHA triglyceride (DHA-TG) for 90 days improved macular function and antioxidant capacity in asymptomatic patients with non-proliferative DR [63]. However, in another study, DHA-TG supplementation for two years did not slow down the progression of non-proliferative DR [64].

DHA is the main major retinal ω3 LC-PUFA. It plays a key role in vision because it is a main component of the outer segments of rod PRs. DHA is involved in the signal transduction process, PR development and rhodopsin activation, among others [65]. ELOVL4 catalyzes the biosynthesis of VLC-PUFAs from DHA or EPA. Both LC-PUFAs and VLC-PUFAs are capable of producing SPMs or elovanoids that present protective effects against oxidative stress and inflammation [66]. In *rd10* mouse retinas, Ruiz-Pastor et al. found a reduced number of fatty acids at P25. Lipidomic analyses revealed an important reduction in ω3 as well as ω6 LC-PUFA, especially for DHA and AA, respectively. The authors proposed that these fatty acids could be essential for PR survival [16].

Most of the beneficial effects of DHA have been associated to their antioxidant properties [67]. In vitro studies showed that DHA protected cells from oxidative damage by promoting endogenous antioxidant defense such as increase activity of antioxidant enzymes (SOD or GPx), or glutathione (GSH) [67]. In the current study, we showed that EFA supplementation reduced the quantity of the markers of oxidative damage, NOX (in BV2 cells), TBARS and CAR (in *rd10* mouse retinas); however, it did not have a significant effect on the antioxidant response markers, except for a slight increase in CAT activity. Some studies indicate that SPMs such as LXs or E-series resolvins (RvE) can regulate redox homeostasis by reducing free radicals including H_2_O_2_, ONOO^−^ or O_2_^−^ [68].

Our findings showed an improvement in retinal function with better ERG recordings and higher sensitivity to bright light in EFA-treated *rd10* mice at P18. Better retinal function is accompanied by better preservation of the retinal structure with lower PR loss and microglia activation at this age. We selected this age to evaluate EFA supplementation (short-term effect) because there is a peak in PR degeneration (rod degeneration) and a significant upregulation of some pro-inflammatory molecules. We and other authors have observed that the inflammatory process starts even before the first peak in degeneration (P18) in *rd10* mouse retinas. However, it is likely that longer-term EFA supplementation may also provide neuroprotection (e.g., against cone degeneration) by reducing the inflammatory process. Thus, long-term nutritional interventions could be a promising alternative until a definitive treatment for RP is found (e.g., gene or cellular therapies). We should evaluate long-term EFA supplementation to confirm our hypothesis.

In this study, we focused on how EFA supplementation could modulate microglia activation and migration in *rd10* mice. Microglia cells are modulators of inflammation [69]. Microglia activation (mainly the M1 phenotype) is a hallmark of neuroinflammation. Several studies have shown that microglia inhibition (M1) ameliorated retinal degeneration in RP and other retinopathies [70,71]. During RP, microglia cells shift to a reactive inflammatory state (M1 phenotype), which is characterized by the acquisition of an amoeboid morphology, phagocytic function and the ability to proliferate and migrate into the ONL, where these cells remove debris of dying PRs, and stressed and living PRs accelerating retinal degeneration [35,72,73]

Homeostatic microglia cells (M0) are activated upon pathologic signals such as harmful stimuli, tissue damage (e.g., gene defect), or free radicals. After activation, M1 is the classical pro-inflammatory phenotype and M2 is the alternative anti-inflammatory phenotype. Subclasses of M2 microglia have been identified including M2a, M2b and M2c phenotypes. These three states overlap in their biochemical roles but differ in their surface and cytosolic markers, mechanism of action and stimuli. Activation of M2a, considered an anti-inflammatory phenotype, is promoted by signals such as IL4 and IL3 and leads to the upregulation of CD206 and RG1, among others. M2a is involved in long-term function for repair and resolution [72,73,74]. Activation of M2b, considered an inflammation regulatory phenotype, is triggered by Fcγ receptors, TLRs and immune complexes [31]. M2b and M1 microglia share similar features but have different responses including regulatory T cell recruitment. M2b expresses COX2, IL10, CD86 and MHCII, among others. Finally, M2c activation, considered an immunosuppressive phenotype, occurs in response to specific anti-inflammatory factors such as, IL-10, TGF-β and glucocorticoids [75,76]. M2c is involved in matrix remodeling, tissue repair and immunoregulation. M2c expresses CD163, TGFβ and IL10 [77] (Table 6).

The activation and polarization of microglia/macrophages is achieved through many, often interconnected, signaling pathways [78,79]. For instance, LPS stimuli activate a signaling pathway leading to an increase in pro-inflammatory cytokines (IL1β, IL6, IL12, TNFα) (M1 and M2b phenotypes) [80,81,82]. M1/M2 stimulation causes transcriptional upregulation of cell surface markers (CD86, CD16/32, MHC-II, CD206, CD163) and intracellular enzymes (iNOS or NOS2, ARG1, etc.). In this study, we corroborated that LPS stimuli induced M1 markers in BV2 cells. Moreover, *rd10* mouse retinas showed microglia activation and migration toward ONL, as well as the confirmed M1 phenotype of this activated microglia. At P18, *rd10* mouse retinas presented a high expression of the M1 markers *Tnfa*, *Il6* and *Il1b* and more CD86-positive microglia than the control retinas. On the other hand, *rd10* mouse retinas showed a lower expression of *Arg1* and *Cd163* than the control retinas.

The polarization of M1 toward the M2 phenotype would ameliorate inflammation and retinal degeneration. For instance, alpha antitrypsin treatment ameliorated retinal degeneration by polarizing microglia toward the M2 phenotype, partly through the regulation of IRF4/IRF8 in *rd1* mice [83]. In our study, we showed that *rd10* mice treated with the EFA supplement presented a high gene expression of the M2 markers *Arg1, Il10, Cd163* and a higher ratio of CD206-positive microglia than untreated *rd10* mice, suggesting an attenuation of M1 markers and a shift from the M1 to the M2 phenotype. According to Table 6, we could propose that EFA supplementation shifted microglia from M1 to M2a or M2c.

Our findings supported those suggesting that SPMs could modulate microglia activation, M2 polarization and even NLRP3 inflammasome activation in neurodegenerative diseases. We previously described an increase in some components of NLRP3 inflammasome in *rd10* mouse retinas at P23. In addition, we showed that the use of antibodies against TNFα ameliorated retinal degeneration and downregulated NLRP3 inflammasome components [84]. MaR1 promoted inflammation resolution by inhibiting chemotaxis, TNFα, IL1β, IL18 levels and NLRP3 inflammasome or NF-kB activation and regulating microglia activation in rodent models of Alzheimer’s disease [85], non-compressive lumbar disc herniation [86] or inflammatory pain [87]. RvD1 ameliorated neuronal death, decreased IL1β, TNFα, malondialdehyde, MDA, and NLRP3 protein in rats with cerebral ischemia/reperfusion injury [88]. MaR1 attenuated M2 markers in macrophages stimulated by LPS [89] or shifted macrophage to a resolving or phenotype induced by β-amyloid [90]. In the retina, RvD1 partially inhibited the upregulation of NLRP3 components in rats with DR [91]. Intravitreal LX4 improved visual function in *rd1* mice [50].

In the current study, we used several markers to study microglia and their activation (Iba1, CD45, CD11b, ARG1, CD206, CD163 and CD86); however, we are aware of our limitations to discriminate resident microglia from monocyte-derived macrophages (MDM). Both cell types share a majority of markers such as CD11b, CD45 or Iba1 [89]. Furthermore, there are overlapping gene patterns for MDM and M2 microglia genes. For instance, perivascular macrophages are CD11b^+^/CD206^high^/CD163+ and resident microglia are CD11b^+^/CD206^low/−^CD163^−^. However, CD206 and CD163 are also expressed by M2-microglia [92]. Currently, there are microglia-specific genes and others for infiltrating cells. However, these genes can vary upon the cell status.

An option to discriminate both of these cell types could be to quantify the amount of proteins such as CD45. Generally, resident microglia express CD11b^+^/CD45^low/int^ and macrophages express CD11b^+^/CD45^high^ [93,94,95]; however, activated microglia could upregulate CD45 as well [96,97,98,99]. In the current study, we analyzed the CD45^low^ subpopulation via flow cytometry of whole retinas; however, we expect to improve the analysis in future studies. We suggested that EFA supplementation affected resident microglia and probably the infiltrating macrophage phenotype; however, we were not able to distinguish subpopulations of immune responders within the retina. Additional studies combining flow cytometry, immunohistochemistry and RNAseq, among others, are needed to discriminate these subpopulations. Regardless, resident microglia and MDM play a cooperative role in the initiation and resolution of inflammation after brain or retinal damage.

For the in vivo experiments, we used a single dose of the EFA supplement (2.82 mg/mL) based on previous studies in mice [100,101,102,103,104]. Most studies related to fatty acid supplementation have focused on DHA or EPA supplementation. To a lesser extent, some studies have focused on a blend of fatty acids derived from fish oils without detailing the exact composition of the blend. In particular, the dose range for DHA and EPA has been quite wide for murine studies. Moreover, there is no a standardized way to indicate the exact dosage of DHA, EPA or others, for all the studies. In some studies, we observed that the EPA and DHA doses ranged from 0.05 to 2 mg/g of bw and from 0.05 to 1.5 mg/g of bw, respectively. In our study, the daily doses of EPA and DHA were within these ranges (0.52 mg/g and 1.15 mg/g, respectively) (Table 2). We believe that the chosen dose was appropriate and within the range of other studies. However, it would be interesting to construct a dose curve to refine the minimum dose with a beneficial effect on retinal degeneration in the future. On the other hand, we do not know the fatty lipid or group of fatty lipids responsible for the protective effect. It is likely that the beneficial effect observed is the result of a synergy between several of the fatty acids present in the EFA supplement. Therefore, we can only assume the overall effect of EFA supplementation and not attribute it to a single molecule. Further studies need to be carried out to determine that aspect.

We are aware of the limitations of our study including the need for a better characterization of microglia phenotypes, discrimination of resident microglia from MDM, a lipidomic analysis of SPMs in *rd10* mouse retinas and a long-term study or a further study of ALOX protein content and cellular localization. For instance, we have not measured the amount of EFA supplementation in the blood or the retina; however, several studies indicate that fatty acid supplementation increases the concentration in these tissues [105,106]. In spite of these limitations, we believe that the current work highlights the importance of SPMs in the progresion of retinal diseases with chronic inflammation such as RP. Furthermore, it opens new therapeutic opportunities based on resolving inflammation with oral supplementation of SPMs such as an EFA supplement.

## 5. Conclusions

RP is a rare disease with a high genetic heterogeneity which makes diagnosis and treatment difficult. Chronic inflammation is a characteristic of RP that would exacerbate retinal degeneration. Therapeutic approaches targeting inflammation will be mutation-independent strategies. Our study proposes nutritional supplementation based on a bioactive formulation containing EPA, DHA and SPM precursors capable of at least partly promoting the resolution of inflammation, and in turn, slowing down the neurodegenerative process. This lipid formulation, named LIPINOVA^®^, is commercially available for human consumption. We believe that this nutritional supplement could have a direct beneficial impact on the quality of life of RP patients. More studies are needed to explore this idea. 

## Figures and Tables

**Table 1:** Essential fatty acid content in EFA supplement batch.

Fatty Acid		g/100 g
Palmitic	C16:0	0.47
Palmitoleic	C16:1 n7	0.28
Hexadecaenoic	C16:4 n1	0.09
Stearic	C18:0	0.78
Oleic	C18:1 n9	1.24
Vaccenic	C18:1 n7	0.51
Linoleic	C18:2 n6	0.19
Linolenic	C18:3 n3	0.11
Stearidonic	C18:4 n3	0.43
Arachidic	C20:0	1.44
Eicosenoic	C20:1 n9	2.78
Gondonic	C20:1 n7	0.38
Arachidonic	C20:4 n6	1.05
Eicosatetraenoic	C20:4 n3	1.15
Eicosapentaenoic	C20:5 n3	18.36
Behenic	C22:0	1.44
Erucic	C22:1 n11	2.78
Adrenic	C22:4 n6	0.46
Docosapentaenoic	C22:5 n6	0.90
Docosapentaenoic	C22:5 n3	6.81
Lignoceric	C24:0	0.44
Docosahexaenoic	C22:6 n3	40.73
Nervonic	C24:1 n9	2.17
Total ω3		67.59
Total ω6		2.14
Total ω9		6.19
SFAs		4.57
MUFAs		10.14
PUFAs		69.13

SFAs: saturated fatty acids; MUFAs: monounsaturated fatty acids; PUFAs: polyunsaturated fatty acids.

**Table 2:** Estimated dose of each fatty acid.

Fatty Acid	mg/g of Body Weight
Palmitic	0.013
Palmitoleic	0.008
Hexadecaenoic	0.003
Stearic	0.022
Oleic	0.035
Vaccenic	0.014
Linoleic	0.005
Linolenic	0.003
Stearidonic	0.012
Arachidic	0.041
Eicosenoic	0.078
Gondonic	0.011
Arachidonic	0.030
Eicosatetraenoic	0.032
Eicosapentaenoic	0.518
Behenic	0.041
Erucic	0.078
Adrenic	0.013
Docosapentaenoic	0.025
Docosapentaenoic	0.192
Lignoceric	0.012
Docosahexaenoic	1.149
Nervonic	0.061
Total ω3	1.906
Total ω6	0.060
Total ω9	0.175
SFAs	0.129
MUFAs	0.286
PUFAs	1.949

**Table 3:** Statistical analysis showing *p*-values of the comparison between control and *rd10* mouse retinas on different postnatal days.

Age-Matched Comparisons ^1^	*Alox5*	*Alox8*	*Alox15*	*Elovl4*
C-*rd10* P13	0.0018	0.0013	0.1510	0.215
C-*rd10* P15	0.0006	0.6846	0.0003	0.0242
C-*rd10* P18	0.0315	0.0677	0.0001	0.0163
C-*rd10* P23	<0.0001	0.0203	<0.0001	<0.0001
C-*rd10* P30	0.208	<0.0001	0.4610	<0.0001
C-*rd10* P44	0.9214	<0.0001	0.0003	<0.0001
C-*rd10* P60	0.1512	0.3495	0.0003	<0.0001

^1^ Parametric data, unpaired *t*-test.

**Table 4:** Percentage of Iba1-positive cells in each nuclear or plexiform layer.

Iba1 + cells (%)	ONL Mean (SEM)	OPL Mean (SEM)	INL Mean (SEM)	IPL Mean (SEM)	GCL Mean (SEM)
C	0.3 (0.3)	25.5 (3.9)	5.3 (3.8)	44.3 (2.4)	24.5 (2.1)
*rd10*	30.7 (2.8)	18.9 (1.2)	10.6 (1.9)	18.8 (1.8)	21.1 (1.0)
*rd10* + EFA	16.0 (3.5)	26.7 (1.0)	4.2 (1.5)	26.0 (1.5)	27.3 (3.8)

**Table 5:** Redox status showing *p*-values of the comparison between control and *rd10* mouse retinas on different postnatal days.

Marker	C Mean (SEM)	*rd10* Mean (SEM)	*rd10* + EFA Mean (SEM)
SOD activity	1.93 (0.17)	2.05 (0.2)	1.67 (0.19)
CAT activity	5.18 (0.53)	7.22 (0.71)	7.73 (0.43) *
TBARS	0.35 (0.07)	1.14 (0.19) ***	0.38 (0.13) ^#^
CAR	3.78 (0.42)	6.06 (0.29) ***	3.55 (0.50) ^##^

One-way ANOVA (for SOD) or Kruskal-Wallis test followed by Tukey’s or Dunn’s multiple comparisons test * *p* < 0.05; *** *p* < 0.001; for differences between control and *rd10* mice or *rd10* + EFA mice; ^#^ *p* < 0.05; ^##^ *p* < 0.01 for differences between *rd10* and *rd10* + EFA mice.

**Table 6:** Some markers of microglia/macrophage subtypes in mice.

Phenotypes	Stimuli	Surface Markers	Intracellular Markers	Functions
M1	IFNγ, LPS, GM-CSF, TNFα	CXCL9, CD86, CD80, CD16/32	IL6, TNFα, iNOS, IL12^high^/IL10^low^	Pro-inflammatory
M2a	IL4, IL13	CD206, IL1-R	CCL17, IL10, ARG1	Anti-inflammatory, repair and resolution
M2b	LPS+ immune complex, ILβ+ immune complex	CD86, CD80	TNFα, IL6, iNOS, COX2, CCL1, IL10^high^/IL12^low^	Regulatory T cell recruitment
M2c	IL10, glucocorticoids	IL4R, CD206, CD163	IL10, TGFβ, Arg1	Immunoregulation, phagocytosis, tissue remodeling

## Data Availability

Data are available in a publicly accessible repository.

## Supplementary Materials

The following supporting information can be downloaded at: https://www.mdpi.com/article/10.3390/antiox12010098/s1, Table S1: List of approved drugs targeting or having an action on target genes ALOX5 and ELOVL4 according to DrugBank database; Table S2: List of circuits affected by ALOX5 and ELOVL4, known drug targets according to the ML model of RP from the KEGG-based mechanistic map; Table S3: Number of each pathway’s circuits in which have an impact drug targets ALOX5 or ELOVL4.
