# Supplementary material for: An SPM-Enriched Marine Oil Supplement Shifted Microglia Polarization toward M2, Ameliorating Retinal Degeneration in rd10 Mice
Source: Antioxidants (Basel). 2022 Dec 30;12(1):98. doi: 10.3390/antiox12010098 (PMC9855087; doi:10.3390/antiox12010098)
Supplement: Supplementary file 1 [file antioxidants-12-00098-s001.zip › antioxidants-1980440-supplementary.pdf]

Table S1. List of approved drugs targeting or having an action over target genes ALOX5 and ELOVL4 according to DrugBank database.

Gene (entrez id)	Drug name	Drugbank Id	Action	Pharmacological action
ALOX5 (240)	Masoprocol	DB00179	inhibitor	yes
	Aminosalicylic acid	DB00233	inhibitor	Unknown
	Mesalazine	DB00244	inhibitor	yes
	Montelukast	DB00471	other/unknown	Unknown
	Diclofenac	DB00586	potentiator	Unknown
	Diethylcarbamazine	DB00711	inhibitor	yes
	Zileuton	DB00744	inhibitor	yes
	Sulfasalazine	DB00795	inhibitor	yes
	Meclofenamic acid	DB00939	inhibitor	yes
	Balsalazide	DB01014	inhibitor	yes
	Minocycline	DB01017	inhibitor	Unknown
	Hyperforin	DB01892	inhibitor	Unknown
	Cannabidiol	DB09061	inhibitor	Unknown
	Morniflumate	DB09285	antagonist, inhibitor	Unknown
	Omega-3 fatty acids	DB11133	substrate	Unknown
	Diacerein	DB11994	inhibitor	Unknown
	Fostamatinib	DB12010	inhibitor	Unknown
	Omega-6 fatty acids	DB13168	substrate	Unknown
	Rhein	DB13174	inhibitor	Unknown
	Isocapent	DB00159	substrate	Unknown
ELOVL4 (6785)	Vayarin	DB09328	substrate	Unknown
	Omega-3-carboxylic acids	DB09568	potentiator	yes

Table S2. List of circuits affected by ALOX5 and ELOVL4 known drug targets according to the ML model of RP KEGG-based mechanistic map.

KEGG pathway	Circuit effector	Function	ALOX5 Relevant	ALOX5 Value	ELOVL4 Relevant	ELOVL4 Value
MAPK signaling pathway	MAPT	Actin binding, neurogenesis	0	-1,99E-05	1	5,50E-05
MAPK signaling pathway	STMN1	Neurogenesis	0	-3,97E-06	1	3,06E-05
MAPK signaling pathway	PLA2G4B	**Lipid degradation**	0	1,77E-06	1	7,93E-05
MAPK signaling pathway	MYC	Transcription regulation	0	-5,06E-06	1	4,24E-05
ErbB signaling pathway	MYC	Transcription regulation	0	-4,21E-07	1	7,04E-06
ErbB signaling pathway	ELK1	Transcription regulation	0	-4,99E-07	1	3,49E-06
Ras signaling pathway	PLA2G4B	**Lipid degradation**	0	-3,07E-06	1	1,17E-05
Ras signaling pathway	ETS1	Transcription regulation	0	6,00E-06	1	1,51E-05
Ras signaling pathway	FASLG	Apoptosis	1	1,36E-05	1	-1,04E-05
Ras signaling pathway	PAK4	Apoptosis	0	3,85E-05	1	0,00016034
Ras signaling pathway	RHOA	Host-virus interaction	0	4,20E-05	1	0,0001648
Ras signaling pathway	RAC1*	Transcription regulation	0	1,50E-05	1	5,25E-05
Ras signaling pathway	PLD1	**Lipid degradation**	0	1,37E-05	1	9,01E-05
Ras signaling pathway	PRKCA	Apoptosis	0	1,50E-05	1	-0,00011985
Ras signaling pathway	D-myo-Inositol 1,4,5-trisphosphate		0	-2,70E-05	1	-0,00026758
Ras signaling pathway	ABL1	Endocytosis	0	-1,19E-05	1	0,00013029
Ras signaling pathway	RAB5A	Endocytosis	0	-1,52E-05	1	0,00018315
Ras signaling pathway	RAC1**	Transcription regulation	0	-5,30E-06	1	0,00010014
Ras signaling pathway	STK4 STK4	Apoptosis	1	7,58E-05	1	5,77E-05
Ras signaling pathway	MLLT4	Cell adhesion	0	-1,43E-05	1	0,00011171

Ras signaling pathway	RASSF5	Apoptosis	1	0,0003972	0	0,00021784
Ras signaling pathway	TIAM1*	Axonogenesis, cell migration	0	7,30E-05	1	0,00053526
Ras signaling pathway	PIK3R5*	Angiogenesis	0	0,0001855	1	-0,00024652
Ras signaling pathway	RAF1*	Apoptosis	1	0,0001763	0	-3,78E-05
Ras signaling pathway	BRAP*	Ubl conjugation pathway	0	5,02E-05	1	0,0001149
Ras signaling pathway	RALGDS*	Transcription regulation	1	0,0001991	0	0,00015961
Ras signaling pathway	PLCE1*	**Lipid degradation**	0	0,0001295	1	-0,00036227
Ras signaling pathway	RIN1*	Endocytosis	0	8,95E-05	1	0,0004064
Rap1 signaling pathway	MAPK1	Cell cycle	0	-4,74E-06	1	3,25E-05
Rap1 signaling pathway	PLCE1	**Lipid degradation**	0	2,34E-05	1	-0,00029782
cGMP-PKG signaling pathway	GTF2I	Transcription regulation	0	-8,80E-05	1	-0,00026383
cAMP signaling pathway	HHIP	Development	0	-8,55E-06	1	-5,87E-05
cAMP signaling pathway	ACOX1	**Fatty acid metabolism**	0	-9,69E-06	1	-3,11E-05
cAMP signaling pathway	ORAI1	Ion transport	0	4,25E-06	1	-5,38E-05
cAMP signaling pathway	BAD	Apoptosis	0	-6,64E-06	1	7,68E-05
cAMP signaling pathway	NFKBIA NFKB1	Host-virus interaction	0	1,36E-05	1	-9,63E-05
cAMP signaling pathway	PLN	Ion transport	0	-4,61E-05	1	-0,00014106
cAMP signaling pathway	RYR2	Ion transport	0	2,52E-05	1	-0,00010022
cAMP signaling pathway	ATP2B1	Ion transport	0	-1,79E-05	1	-9,22E-05
cAMP signaling pathway	CACNA1C	Ion transport	0	1,12E-05	1	-9,66E-05

cAMP signaling pathway	PDE3A	Angiogenesis	0	5,18E-06	1	-5,38E-05
cAMP signaling pathway	LIPE	**Cholesterol metabolism**	0	-2,89E-05	1	-8,53E-05
cAMP signaling pathway	ATP1B4 FXYD1	Ion transport	0	-3,61E-05	1	-0,00011299
cAMP signaling pathway	AKT3	Glycogen biosynthesis	0	3,57E-06	1	-2,01E-05
cAMP signaling pathway	Diacylglycerol		0	1,73E-05	1	-0,00010826
cAMP signaling pathway	D-myo-Inositol 1,4,5-trisphosphate		0	1,73E-05	1	-0,00010826
cAMP signaling pathway	AFDN	Cell adhesion	0	9,68E-06	1	-4,30E-05
cAMP signaling pathway	MAPK8	Biological rhythms	0	-2,44E-05	1	-8,99E-05
Chemokine signaling pathway	MAPK1	Host-virus interaction	1	2,81E-05	0	1,10E-05
FoxO signaling pathway	FASLG	Apoptosis	1	5,81E-17	0	-4,10E-17
Sphingolipid signaling pathway	MAPK1	Apoptosis	0	1,13E-05	1	2,09E-05
Phospholipase D signaling pathway	MAPK1	Host-virus interaction	0	3,87E-06	1	1,14E-05
PI3K-Akt signaling pathway	GYS1	Glycogen biosynthesis	0	6,70E-07	1	-3,42E-06
PI3K-Akt signaling pathway	G6PC	Gluconeogenesis	0	-9,55E-07	1	-2,58E-06
PI3K-Akt signaling pathway	FASLG	Apoptosis	1	2,62E-06	1	-2,27E-06
PI3K-Akt signaling pathway	TP53	Cell cycle	0	-2,00E-06	1	4,16E-06
PI3K-Akt signaling pathway	MYB	Transcription regulation	0	1,94E-06	1	3,40E-06

PI3K-Akt signaling pathway	BCL2	Apoptosis	0	1,53E-06	1	-4,96E-06
PI3K-Akt signaling pathway	EIF4B	Initiation factor	1	1,09E-05	0	6,58E-06
PI3K-Akt signaling pathway	RPS6	Cell cycle	1	1,34E-05	0	1,05E-05
PI3K-Akt signaling pathway	NOS3	Translation regulation	0	7,89E-06	1	-1,13E-05
PI3K-Akt signaling pathway	PRKCA	Apoptosis	0	2,16E-05	1	-4,36E-05
PI3K-Akt signaling pathway	PKN3	Transcription regulation	1	3,74E-05	1	-4,32E-05
Longevity regulating pathway - mammal	CAT	Detoxification	0	-1,85E-05	1	-0,0001784
Longevity regulating pathway - mammal	RPS6KB1	Translation regulation	0	-5,80E-06	1	0,00016615
Hedgehog signaling pathway	GLI1*	Transcription regulation	0	3,20E-06	1	1,23E-05
Hedgehog signaling pathway	HHIP		0	5,49E-06	1	-3,05E-05
Axon guidance	CFL1	Actin formation	0	-1,47E-05	1	-4,92E-05
Gap junction	GJA1 GJA1***	Cell adhesion	0	-0,000188	1	0,00048918
Gap junction	GJA1 GJA1	Cell adhesion	0	-7,88E-10	1	-1,89E-09
Gap junction	GJA1 GJA1*	Cell adhesion	0	-7,88E-10	1	-1,89E-09
Gap junction	GJA1 TJP1	Cell adhesion	0	-7,58E-10	1	-2,02E-09
Natural killer cell mediated cytotoxicity	TNF	Acute inflammatory response	1	2,50E-05	0	-3,86E-06
Natural killer cell mediated cytotoxicity	CSF2	Neurogenesis	1	8,28E-06	0	-2,41E-06
Natural killer cell mediated cytotoxicity	FAS	**Fatty-acid biosynthesis**	1	0,0002945	1	-0,00020267
Natural killer cell mediated cytotoxicity	TNFRSF10D	Apoptosis	1	0,0002182	1	-0,00030359

Fc epsilon RI signaling pathway	PLA2G4B	**Lipid degradation**	0	9,71E-08	1	3,71E-07
Fc gamma R-mediated phagocytosis	CFL1	Actin binding, synapse, neurogenesis	1	-2,96E-05	0	1,13E-05
GABAergic synapse	GABRA1 GPHN	Chloride channel	0	-0,000311	1	0,00071121
Regulation of actin cytoskeleton	ACTB ARPC5	Actin binding	0	-2,31E-09	1	2,50E-08
Regulation of actin cytoskeleton	CFL1 ACTB	Actin binding, synapse, neurogenesis	0	4,80E-06	1	-1,72E-05
Regulation of actin cytoskeleton	MYL12B MYH9 ACTB	Cell shape	0	4,44E-12	1	-6,57E-12
Regulation of actin cytoskeleton	PXN	Cell adhesion	0	3,30E-06	1	-1,49E-05
Regulation of actin cytoskeleton	VCL*	Cell adhesion	1	8,77E-11	1	-1,18E-10
Regulation of actin cytoskeleton	ACTN4	Protein transport	0	9,15E-11	1	-1,16E-10
Regulation of actin cytoskeleton	MSN	Cell shape	1	1,07E-10	0	-8,89E-11
Insulin signaling pathway	ELK1	Transcription regulation	0	-9,13E-08	1	8,13E-07
Melanogenesis	TYR*	Melanin biosynthesis	0	-1,01E-07	1	3,08E-06
Melanogenesis	TYRP1	Melanin biosynthesis	0	5,01E-07	1	3,21E-06
Melanogenesis	DCT	Melanin biosynthesis	0	-4,80E-07	1	2,49E-06
Oxytocin signaling pathway	MEF2C	Neurogenesis	0	1,22E-05	1	-8,31E-05
Oxytocin signaling pathway	CCND1	Cell cycle	0	-2,62E-05	1	0,00015234

* Each star refers to the number pf times the referred gene acts as an effector in that pathway.

0 means that the target is not relevant in the circuit, while 1 means that the target gene has an impact, therefore is relevant, for the prediction of the circuit activity.

Table S3. Number of each pathway's circuits in which have an impact drug targets ALOX5 or ELOVL4

KEGG pathway	Number of relevant circuits		Total number of circuits	Percentage of pathway	
	**ALOX5**	**ELOVL4**		**ALOX5**	**ELOVL4**
MAPK signaling pathway	0	4	28	0,00	14,29
ErbB signaling pathway	0	2	18	0,00	11,11
Ras signaling pathway	5	19	32	**15,63**	**59,38**
Rap1 signaling pathway	0	2	14	0,00	14,29
cGMP-PKG signaling pathway	0	1	22	0,00	4,55
cAMP signaling pathway	0	17	35	0,00	**48,57**
Chemokine signaling pathway	1	0	13	7,69	0,00
FoxO signaling pathway	1	0	30	3,33	0,00
Sphingolipid signaling pathway	0	1	11	0,00	9,09
Phospholipase D signaling pathway	0	1	4	0,00	25,00
PI3K-Akt signaling pathway	4	9	28	14,29	**32,14**
Longevity regulating pathway - mammal	0	2	13	0,00	15,38
Hedgehog signaling pathway	0	2	15	0,00	13,33
Axon guidance	0	1	31	0,00	3,23
Gap junction	0	4	5	0,00	**80,00**
Natural killer cell mediated cytotoxicity	4	2	11	**36,36**	18,18
Fc epsilon RI signaling pathway	0	1	6	0,00	16,67
Fc gamma R-mediated phagocytosis	1	0	12	8,33	0,00
GABAergic synapse	0	1	7	0,00	14,29
Regulation of actin cytoskeleton	2	6	12	**16,67**	**50,00**
Insulin signaling pathway	0	1	15	0,00	6,67
Melanogenesis	0	3	7	0,00	**42,86**
Oxytocin signaling pathway	0	2	22	0,00	9,09
